# Financial incentives for maintaining postpartum smoking abstinence: 3–4‐year follow‐up of the Financial Incentives for Prevention of Postpartum Return to Smoking (FIPPS) randomised controlled trial

**DOI:** 10.1111/add.70505

**Published:** 2026-07-14

**Authors:** Michael Ussher, Catherine Best, Sarah Lewis, Linda Bauld, Jennifer McKell, Sophie Orton, Sue Cooper, Tim Coleman

**Affiliations:** ^1^ Institute for Social Marketing and Health University of Stirling Stirling UK; ^2^ Department of Population Health and Policy, City St George's University of London London UK; ^3^ Faculty of Medicine and Health Sciences University of Nottingham Nottingham UK; ^4^ Usher Institute and Behavioural Research UK University of Edinburgh Edinburgh UK; ^5^ Centre for Academic Primary Care University of Nottingham Nottingham UK

**Keywords:** financial incentives, intervention, maintenance, postpartum, pregnancy, randomized controlled trial, relapse prevention, smoking, smoking abstinence, vouchers

## Abstract

**Background and aims:**

We followed up a financial incentives trial aimed at maintaining smoking cessation postpartum, which showed some positive effects at one year, to assess sustained abstinence at 3–4 years postpartum.

**Design, setting and participants:**

Secondary analysis of longer‐term outcomes of a pragmatic, multi‐centre, three‐arm randomized controlled trial, recruiting 462 postpartum women in England, aged ≥16 years, having stopped smoking during pregnancy with financial incentives, validated as abstinent at end of pregnancy or early postpartum. Interventions: (1) usual care (UC); (2) UC plus up to £60 of voucher incentives offered to participants and £60 offered to significant‐other supporters, over 3 months postpartum, contingent upon validated abstinence (‘3‐months incentives’); or (3) UC plus ‘3‐months incentives’ plus £180 of vouchers offered to participants over 9 months postpartum (leading to a maximum of £240 of incentives for participants, plus £60 for significant others), contingent upon validated abstinence (‘12‐months incentives’). Participants who did not achieve sustained abstinence at one year were included as non‐abstainers but were not followed‐up.

**Measurements:**

Primary outcome: biochemically validated abstinence at 3–4 years postpartum. Statistical significance was set at *P* < 0.017 to account for multiple comparisons.

**Findings:**

Among the full trial sample, 63% (289/462) of participants had self‐reported smoking status at 3–4 years postpartum; 19% (88/462) reported as abstinent, of whom 64% (56/88) underwent biochemical validation with none misclassified. Primary outcome abstinence was 16.4% (26/159) for 12‐months incentives, 12.3% (19/154) for 3‐months incentives and 7.4% (11/149) for UC. Adjusted odds ratios (OR) (95% confidence interval) for 12‐months vs. UC: OR 2.51 (1.18–5.31), *P* = 0.016, and for 3‐months vs. UC: 1.81 (0.83–3.98), *P* = 0.138. In a fully adjusted model, the difference between 12‐month incentives and UC was no longer statistically significant: OR 2.44 (1.11–5.36), *P* = 0.027, using the Bonferroni correction (*P* < 0.017). Bayes factors indicated that for 12‐months vs. UC there was good evidence for the alternative hypothesis and for 3‐months vs. UC there was inconclusive evidence for the null or alternative hypothesis.

**Conclusions:**

Up to £300 of voucher incentives offered over 12 months postpartum may be effective for maintaining smoking abstinence at 3–4 years postpartum compared with usual care. Up to £120 of incentives over 3 months postpartum does not appear to be beneficial compared with usual care.

## INTRODUCTION

Smoking prevalence is higher among women of reproductive age compared with the general population [1]. Many people cease smoking in pregnancy, however, up to three‐quarters may relapse within 6 months of giving birth [2]. There are also marked health inequalities, as those with lower incomes are more likely to relapse [3]. Preventing postpartum relapse to smoking is a public health priority, yet there are no evidence‐based interventions [4] and new approaches are needed.

During pregnancy, offering financial incentives for smoking cessation is highly effective and cost‐effective [5, 6, 7, 8] as well as being generalisable across multiple service models [5]. We conducted the first large randomised controlled trial [Financial Incentives for Prevention of Postpartum Return to Smoking (FIPPS)] to evaluate whether postpartum financial incentives can aid maintenance of smoking cessation up to 1 year postpartum [9, 10]. We recruited 462 postpartum women (≥16 years old), from four English, National Health Service (NHS), stop smoking services. We conducted a pragmatic, three‐arm trial, comparing (i) usual care (UC) with (ii) UC plus up to £60 of financial voucher incentives offered to participants and £60 offered to an optional significant‐other‐supporter, over 3 months postpartum, contingent on validated smoking abstinence (‘3 months incentives’) or (iii) UC plus ‘3 months incentives’ plus £180 of vouchers offered to participants over 9 months postpartum, contingent on abstinence (‘12 months incentives’). The primary outcome was biochemically verified smoking abstinence at 1 year postpartum. Abstinence rates were approximately twice as high for 12 months incentives (63/159 [39.6%]) vs. 3 months [33/154 (21.4%) (adjusted OR 95% CI = 2.41 [1.46–3.96], *P* = 0.001). The difference in abstinence between 12 months and UC [42/149 (28.2%)] approached significance [OR = 1.67 (1.04–2.70), *P* = 0.035, with Bonferroni correction *P* < 0.017]. There was no evidence for different abstinence rates for 3 months incentives vs. UC [0.69 (0.41–1.17), *P* = 0.174]. As the follow‐up for the primary outcome coincided with the end of the 12 months incentives intervention, it is important to examine whether the benefit of this intervention is maintained once incentives are withdrawn. Sustained postpartum smoking cessation has significant health benefits for the mother, as most new mothers are young enough to minimise long‐term harm, especially from cancers and cardiovascular disease [11]. Sustained maternal smoking cessation also has benefits for the infant in terms of reduced second‐hand smoke exposure, which is associated with child ill health and mortality [12] and reduced likelihood of becoming smokers themselves in later life [13].

We are aware of only two previous studies of interventions to maintain postpartum smoking cessation that have followed up participants beyond 12 months postpartum [14, 15]. We conducted the first study to examine long‐term rates of smoking abstinence, at between 3 and 4 years postpartum, in a trial of financial incentives for maintaining postpartum smoking cessation.

## METHODS

### Study design and participants

This follow‐up study was approved by the North‐West‐Liverpool Central Research Ethics Committee (ref: 16/NW/0838). We conducted a secondary analysis of longer‐term outcomes of the FIPPS study, which was a pragmatic, multicentre, phase III, parallel‐group, three‐arm, individually randomised, controlled trial. The protocol and main findings for the trial are published [9, 10] (see Data S1 and S2). The protocol for this follow‐up study is reported at https://osf.io/jp2q9 (registered on 26 February 2024, before data collection commenced). We aimed to follow‐up 267 participants, including 187 who self‐reported abstinence from smoking at 1 year postpartum (138 biochemically validated as abstinent plus 49 with self‐report only) and 80 who were not contactable at 1 year postpartum. A further 29 participants who were not contactable at 1 year were not followed up as they had declared that they did not wish to take part in further research. Participants who reported smoking at 1‐year follow‐up were not followed up at 3 to 4 years as the primary outcome was for lapse‐free abstinence, therefore, these individuals were counted as having relapsed.

### Procedures

NHS pregnancy stop smoking advisors at the Hospital Trusts that recruited to the trial, posted a letter to the 267 eligible participants, inviting them to take part in the follow‐up study (Northern Care Alliance NHS Foundation Trust *n* = 104, Manchester University NHS Foundation trust *n* = 61, Bolton NHS Foundation trust *n* = 55, Tameside and Glossop Integrated Care NHS Foundation Trust *n* = 47). To maximise engagement, where possible, the letter was sent and followed up by an advisor who had assessed the woman's smoking status during the trial. Those conducting the follow‐ups were aware of group allocation. The letter included a summary of the trial findings, a participant information sheet and a consent form. The letter explained that the advisor would call in the next week to invite them to participate and to answer questions. They could choose to contact the advisor, including declining participation.

During the call, verbal consent was obtained by the advisor who signed the consent form on behalf of the participant. Advisors, who were specially trained for the study, completed the follow‐up questionnaire with participants. For participants reporting continuous abstinence from smoking since their last trial assessment, the advisor arranged a visit to confirm abstinence, via an exhaled carbon monoxide (CO) reading. Participants were offered a £20 high street shopping voucher for completing the follow‐up.

Researchers at the University of Stirling added data to a secure database and conducted data monitoring (see Data S3). Preparation of participant materials included two patient and public involvement and engagement (PPIE) representatives who had smoked during pregnancy. There was additional PPIE representation on the Trial Steering Committee.

### Measures

Consistent with the primary outcome for the main trial findings [10], the primary outcome for the 3‐ to 4‐year follow‐up was self‐reported, sustained (i.e. lapse‐free), smoking abstinence since the last trial assessment, biochemically validated by an exhaled CO reading of less than 8 p.p.m. A cut‐off of less than 8 p.p.m. is standard and consistent with the Russell Standard [17]. We deviated from the Russell Standard in that we required reports of lapse‐free abstinence, rather than allowing lapses with up to five cigarettes in total [17]. This was because participants were recruited from a programme offering financial incentives based on reports of lapse‐free smoking abstinence during pregnancy and we wished to remain consistent with that programme.

Consistent with the secondary outcomes for the main trial findings [10], secondary outcomes included the proportion of women self‐reporting: smoking abstinence; use of nicotine replacement therapy, heat‐not‐burn products or vaping in the last week; interest in stopping vaping; a partner who smokes; smokers living in their home; breastfeeding; subsequent pregnancies and use of smoking cessation support (see follow‐up questionnaire: Data S4).

### Data analysis

Analyses followed the same plan as for the main analysis at the 1 year postpartum follow‐up (https://osf.io/nckj9/) using Stata (StataCorp, release 17) and SPSS version 29. Hypothesis tests were two‐sided. The intention‐to‐treat population was defined as ‘all participants who were randomly assigned to the study and eligible to participate’.

Baseline data were descriptively summarized by study group for all trial participants, and for participants who provided data for the primary outcome at 3 to 4 years [18]. We used χ^2^ tests to compare follow‐up rates between study groups. For analysis of biochemically validated smoking outcomes, consistent with the Russell Standard [17], participants who were included in the analysis were counted as smokers if their smoking status at the final follow‐up could not be determined.

Analysis for the primary outcome used a mixed‐effects logistic regression model with randomised treatment group as a fixed effect, and recruiting site adjusted for as a random effect (random intercept only, to control for non‐independence of observations within sites) [16], with pairwise comparisons between treatment groups, using a significance level of *P* < 0.017 (Bonferroni correction for multiple comparisons [19]). Bayes factors (BFs) were produced using an online calculator (https://users.sussex.ac.uk/~dienes/inference/Bayes.htm) to examine whether there was evidence for the alternative (H1) or null (H0) hypothesis. Usual conventions were applied (i.e. good evidence for H1 over H0 if BF >3; good evidence for H0 over H1 if BF <1/3; otherwise, inconclusive evidence). We set the hypothesized OR to 1.5, but also examined the effect of varying the lower bound from 1.2 to 2, using a half‐normal prior distribution. We also looked for a linear trend in abstinence across the three groups, from UC to 12‐month incentives (0 = UC, 1 = 3 months incentives, 2 = 12 months incentives, significance *P* < 0.05).

Following recommended practice [20], we conducted secondary analyses for the primary outcome, adjusting for pre‐specified baseline covariates that were likely to be strongly prognostic for the primary outcome (i.e. education: A‐level or equivalent or higher vs. lower qualifications, cigarette consumption before pregnancy, depression score [21] and age). These covariates were selected based on a review of predictors of postpartum smoking status [3]. We also considered a sensitivity analysis to examine effects on the primary outcome for other baseline variables that had both a marked difference between groups and were associated with smoking status, but no such variables were identified. For the primary outcome, sensitivity to missing data was assessed with three methods: complete case analysis; multiple imputation by chained equations; and a pattern‐mixture model to assess sensitivity to deviations from the missing data assumptions. A further secondary analysis, with the same adjustments as for the primary outcome, entailed group comparisons for self‐reported smoking status. Descriptive statistics were produced for other secondary outcomes, including validated abstinence rates at 3 to 4 years postpartum and exclusively for those who had been validated as abstinent at 1 year postpartum (i.e. excluding those who at 12 months were not contactable or had self‐report only for smoking abstinence). No outcome data were excluded.

## RESULTS

**FIGURE 1:**
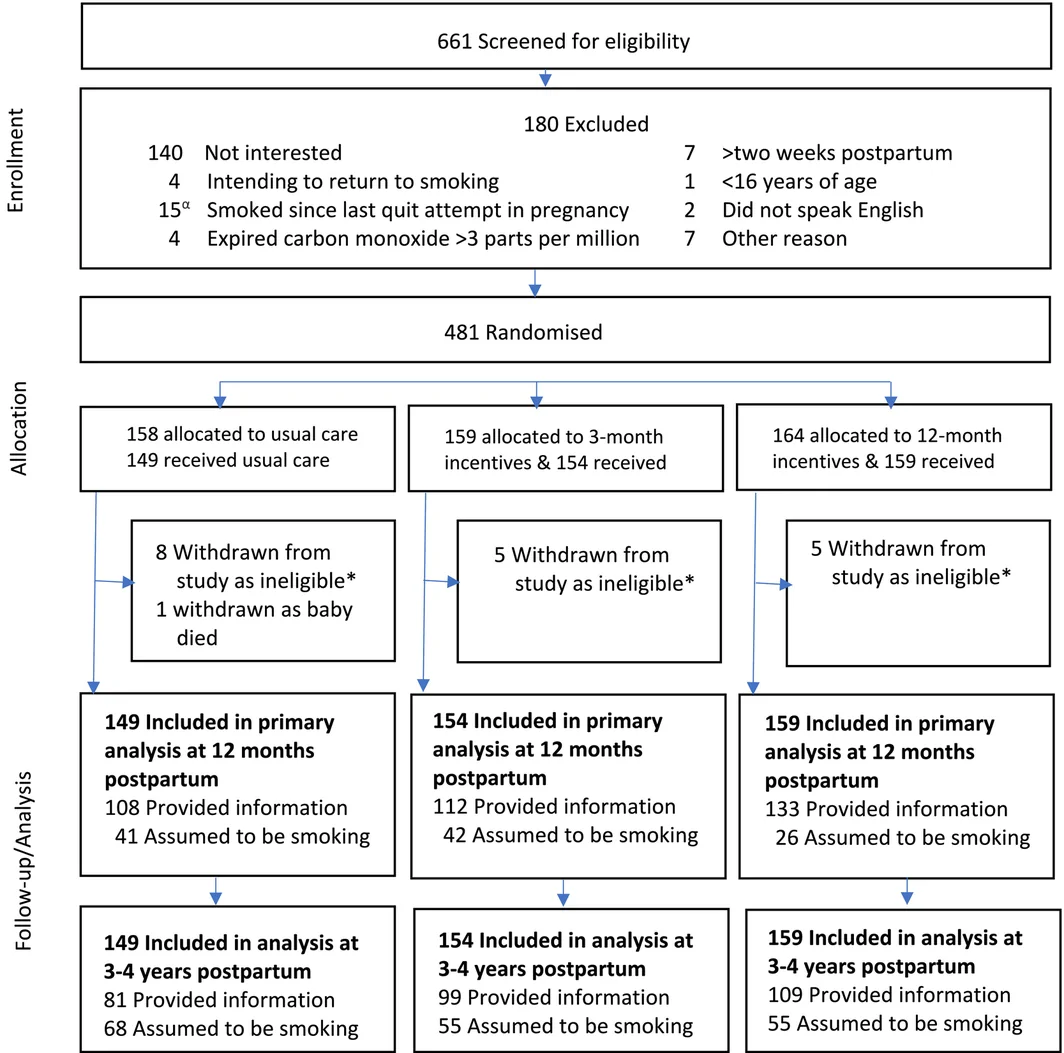
Trial profile of potential participants, participants who were enrolled and randomly assigned to a group and participants whose data was analysed*.* ^α^This eligibility criteria was subsequently replaced with ‘self‐reported not smoking a single puff of a cigarette for at least 4 weeks’, to include those who had had some earlier lapses, but were abstinent ≥4 weeks. Those recruited before this amendment were all abstinent for ≥4 weeks and following this amendment no one was excluded for having smoked in the last 4 weeks.

From 1 March 2024 to 30 June 2024, among 267 women we attempted to follow‐up, 46% (123/267) provided smoking status data at 3 to 4 years (12 months incentives: 57.6% (57/99), 3 months incentives: 48.2% (40/83), UC: 30.6% (26/85), *P* = 0.001). Overall, the mean follow time was 3.7 years postpartum. No contact could be made with 136 participants (53 had also been uncontactable at 1 year postpartum) and eight declined to take part. Including those who had previously been confirmed as smoking at 1 year postpartum (and were, therefore, recorded as having relapsed at 3–4‐years), overall at 3 to 4‐years, 62.5% (289/462) had follow‐up data for self‐reported smoking status [12 months: 68.6% (109/159), 3 months: 64.3% (99/154), UC: 54.4%: (81/149), *P* = 0.032)]; 37.5% (173/462) were assumed to be smoking (Figure 1).

The mean age of the total sample (*n* = 462) was 28.3 years; 87.2% were recruited in late pregnancy and 12.8% joined in early postpartum (Table 1). For 95.5% of participants, the intervention was delivered by a midwife‐led stop smoking service. At baseline, use of nicotine replacement therapy was reported by 13.2% of participants and e‐cigarette use by 28.6%. Baseline characteristics of those followed up at 3 to 4 years were largely similar to the whole sample (Table 1). At 3 to 4 years, the study groups had very similar baseline characteristics (Table 1), except that, compared with the other groups, the 12 months incentives group reported higher levels of education and was more likely to report an intention to breastfeed for at least 4 months; the incentives groups reported more use of nicotine replacement therapy and e‐cigarettes than UC; and the UC group was more likely to report having a partner who smokes and living with smokers.

**TABLE 1:** Baseline characteristics of all participants in analysis and participants followed up at 3 to 4 years by study group.

	All participants in analysis			Total (*n* = 462)	Participants followed up at 3–4 years		
	Usual care (*n* = 149)	3‐month incentives (*n* = 154)	12‐month incentives (*n* = 159)		Usual care (*n* = 26)	3‐month incentives (*n* = 40)	12‐month incentives (*n* = 57)
Demographic characteristics							
Maternal age in years, mean (SD)	27.7 (5.6)	28.6 (5.4)	28.6 (5.9)	28.3 (5.7)	29.2 (5.8)	29.3 (5.3)	29.6 (5.9)
A‐level, degree or equivalent	72 (48.3) m[2]	77 (50.0)	93 (58.5)	242 (52.4)^m[2]^	16 (64.0)^m[1]^	19 (47.5)	39 (68.4)
White ethnicity	140 (94.0)	142 (92.2)	145 (91.2)	427 (92.4)	26 (100.0)	34 (85.0)	53 (93.0)
Type of stop smoking service							
Midwife led	144 (96.6)	145 (94.2)	152 (95.6)	441 (95.5)	25 (96.2)	39 (97.5)	55 (96.5)
Smoking history							
Pre‐pregnancy cigarettes smoked a day, mean (SD)	12.9 (7.2)^m[1]^	13.5 (6.7)	12.4 (6.8)^m[2]^	12.9 (6.9)^m[3^]	12.4 (7.0)	12.8 (7.3)	12.5 (6.5)
Weeks of continuous smoking abstinence, mean (SD)	22.8 (6.4)	22.1 (6.6)^m[1]^	23.3 (6.3)^m[1]^	22.7 (6.5)^m[2]^	24.7 (6.3)	21.9 (5.6)	23.3 (6.1)
Expired CO level p.p.m., mean (SD)	1.1 (0.8)	1.2 (0.7)^m[1]a^	1.2 (0.8)^m[1]b^	1.2 (0.8)^m [2]^	1.0 (0.7)	1.0 (0.7)^m[1]a^	1.3 (0.8)
Very or extremely confident in maintaining smoking abstinence	107 (71.8)^m[1]^	99 (64.3)	110 (69.2)	316 (68.4)^m[1]^	17 (65.4)	25 (62.5)	38 (66.7)
Uses nicotine replacement therapy	15 (10.1)	22 (14.3)	24 (15.1)	61 (13.2)	1 (3.8)	7 (17.5)	8 (14.0)
Uses electronic‐cigarettes	36 (24.2)	51 (33.1)	45 (28.3)	132 (28.6)	6 (23.1)	17 (42.5)	19 (33.3)
Uses heat‐not‐burn	4 (2.7)	2 (1.3)	4 (2.5)	10 (2.2)	1 (3.8)	0	1 (1.8)
Use of smoking cessation support beyond that in trial	0	0	0	0	0	0	0
Partner smokes	47 (31.5)	49 (31.8)	49 (30.8)	145 (31.4)	8 (30.8)	9 (22.5)	14 (24.6)
Living with smokers	49 (32.9)	56 (36.4)	57 (35.8)	162 (35.1)	8 (30.8)	8 (20.0)	16 (28.1)
Has significant‐other‐supporter	66 (44.3)	72 (46.8)	86 (54.1)	224 (48.5)	15 (57.7)	19 (47.5)	32 (56.1)
Pregnancy							
Weeks pregnant, mean (SD)^c^	36.4 (1.0)	36.3 (1.0)	36.5 (1.2)	36.4 (1.1)	36.6 (1.2)	36.5 (1.2)	36.5 (1.2)
Days postpartum, mean (SD)^d^	8.1 (5.2)	8.4 (4.1)	7.6 (4.1)	8.0 (4.4)	7.0 (3.6)	7.4 (4.5)	6.5 (5.6)
Parity, mean (SD)	1 (1.1)^m[2]^	1.2 (1.5)	1.0 (1.1)	1.1(1.2)^m [2]^	0.9 (1.1)^m[1]^	1.5 (1.9)	1.1 (1.1)
Intend to bottle and breastfeed	25 (16.8)	23 (14.9)	30 (18.9)	78 (16.9)	5 (19.2)	5 (12.5)	11 (19.3)
Intend to breastfeed only	77 (51.7)	75 (48.7)	78 (49.1)	230 (49.8)	13 (50.0)	20 (50.0)	31 (54.4)
Intend to breastfeed for ≥4 months	54 (36.2)	48 (31.2)	48 (30.2)	150 (32.5)	7 (38.9)	9 (36.0)	23 (56.1)
Alcohol Use Disorders Identification‐Consumption							
Risk drinking (score ≥5)	2 (1.3)	4 (2.6)	3 (1.9)	9 (1.9)	0	1 (3.8)	2 (3.5)
EDS							
Overall EDS scores, mean (SD)	6.0 (5.0)^m [4]^	5.4 (5.1)^m^ [5]	5.3 (4.7)^m^ [2]	5.6 (4.9)^m^ [11]	6.2 (5.8)^m^ [2]	5.4 (4.7)	5.5 (4.8)^m^ [1]
Major depressive disorder (EDS ≥11)	29 (19.5)^m[4]^	29 (18.8)^m^ [5]	27 (17.0)^m^ [2]	85 (18.4)^m^ [11]	6 (25.0)^m^ [2]	8 (20.0)^m^ [5]	12 (21.4)^m^ [1]

*Note*: Data are *n* (%) of participants unless otherwise stated. Characteristics of those who were followed up at 3–4 years are largely similar to those of the whole trial sample, except the 12‐month group reported more women saying they intend to breastfeed for at least 4 months.

Abbreviations: CO, carbon monoxide; COVID‐19, coronavirus disease 2019; EDS, Edinburgh Depression Scale; ^m^[], missing data [number missing]; p.p.m., parts per million.

^a^ Missing because of medical condition.

^b^ Missing because of COVID‐19 distancing.

^c^ Relates to those recruited in pregnancy.

^d^ Relates to those recruited postpartum.

Among the 19.0% (88/462) of participants reporting smoking abstinence at 3 to 4 years, 63.6% (56/88) underwent CO verification. Rates of undergoing this verification were similar across the groups (Table 2). There were few differences in the characteristics of those who did and did not undergo verification (Data S5). All those undergoing verification were validated as abstinent.

**TABLE 2:** Primary outcome derivation and primary analysis, by study group for follow‐up at 3 to 4 years postpartum.

	Usual care (UC) (*n* = 149)	3‐month incentives (*n* = 154)	12‐month incentives (*n* = 159)	Total (*n* = 462)
Self‐reported smoking status^a^				
Abstinent	17/149 (11.4)	30/154 (19.5)	41/159 (25.8)	88/462 (19.0)
Smoking	64/149 (43.0)	69/154 (44.8)	68/159 (42.8)	201/462 (43.5)
Missing because of no contact	64/149 (43.0)	53/154 (34.4)	48/159 (30.2)	165/462 (35.7)
Missing as declined to take part	4/149 (2.7%)	2/154 (1.3)	2/159 (1.3)	8/462 (1.7)
Self‐reported as abstinent and underwent exhaled CO verification test				
Yes	11/17 (64.7)	19/30 (63.3)	26/41 (63.4)	56/88 (63.6)
No	6/17 (35.3)	11/30 (36.7)	15/41 (36.6)	32^c^/88 (36.4)
Biochemically verified smoking status (primary analysis)				
Abstinent	11^d^/149 (7.4)	19^e^/154 (12.3)	26^f^/159 (16.4)	56/462 (12.1)
Smoking	138/149 (92.6)	135/154 (87.7)	133/159 (83.6)	406/462 (87.9)

*Note*: Group comparisons for primary analysis^g^ OR (95% CI).

12‐month v. 3‐month incentives: OR = 1.38 (0.73–2.64), risk difference 4% (95% CI = 0%–12%), *P* = 0.325.

12‐month incentives vs. UC: OR = 2.51 (1.18–5.31); risk difference 9% (1%–17%), *P* = 0.016.

3‐month incentives vs. UC: OR = 1.81 (0.83–3.98), risk difference 5% (0%–11%), *P* = 0.138.

*Notes*: Data are *n* (%) of participants.

Abbreviations: CO, carbon monoxide; UC, usual care.

^a^ Group comparisons for self‐reported smoking status, fully adjusted ORs (95% CIs): 12‐month vs. 3‐month incentives 1.30 (0.67–2.55), *P* = 0.437; 12‐month incentives vs. UC = 2.60 (1.17–5.80), *P* = 0.019; 3‐month incentives vs. UC = 1.99 (0.87–4.57), *P* = 0.102. Adjusted for education, age, cigarette consumption before pregnancy, depression, smokers in home, partner smoking, use of a significant‐other supporter and intention to breast feed.

^b^ 8 participants had moved out of the area.

^c^ Eleven participants followed up at 3–4 years had missing data at the 12‐month follow‐up:

^d^ One participant could not be contacted.

^e^ Five participants had self‐report only and 2 could not be contacted.

^f^ Three participants had self‐report only.

^g^ Fully adjusted ORs (95% CIs): 12‐month vs. 3‐month incentives 1.25 (0.65–2.44), *P* = 0.496; 12‐month incentives vs. UC = 2.44 (1.11–5.36), *P* = 0.027; 3‐month incentives vs. UC = 1.94 (0.85–4.40), *P* = 0.102. Adjusted for education, age, cigarette consumption before pregnancy and depression.

### Primary outcome

Table 2 presents the primary outcome analysis, adjusting for site. Validated abstinence was higher for 12 months incentives (16.4%) compared with UC (7.4%); OR = 2.51 (95% CI = 1.18–5.31); risk difference 9% (95% CI = 1%–17%), *P* = 0.016. The difference in validated abstinence between 12 months incentives and 3 months incentives (12.3%) was not significant; (OR = 1.38 (0.73–2.64), risk difference 4% (0%–12%), *P* = 0.325. Nor was the difference significant between 3 months incentives and UC; OR = 1.81 (0.83–3.98), risk difference 5% (0%–11%), *P* = 0.138). In a fully adjusted model, the difference between 12‐month incentives and UC was no longer significant (*P* = 0.027), using the Bonferroni correction (*P* < 0.017) (Table 2). BFs indicated that for 12 months incentives vs. UC there was good evidence for H1 (BF >3). For a hypothesized effect size of 1.5, the BF for 12 months vs. 3‐month incentives was 1.30 and for 3 months incentives vs. UC it was 2.08, implying inconclusive evidence for H0 or H1. In addition, there was a significant linear trend in abstinence across the three groups OR = 1.56 (1.09–2.23), *P* = 0.016 and the linear model was a better fit for the data vs. the dummy coded model (see Data S6).

We made the *a priori* decision to fit a model with centre as a random effect [20]. Despite negligible clustering, we retained this model, as it was pre‐specified. Applying an alternative model, including centre as a fixed effect there was no impact on study findings nor was there a site × treatment interaction.

In a complete case analysis, the comparison between 12 months incentives and UC was no longer statistically significant [OR = 2.02 (0.93–4.40), *P* = 0.076], and the comparisons between 12 months and 3 months incentives and 3 months vs. UC remained non‐significant [OR = 1.32 (0.67–2.57), *P* = 0.422; OR = 1.54 (0.68–3.47), *P* = 0.300, respectively]. Similarly, there was no difference between the groups when using multiple imputation of chained equations [12 months vs. 3 months: OR = 1.18 (0.64–2.17), *P* = 0.592; 12 months vs. UC: OR = 1.93 (0.94–3.97), *P* = 0.075; 3 months vs. UC: OR = 1.63 (0.77–3.44), *P* = 0.199] (see Data S7). Pattern‐mixture modelling showed that for none of the comparisons between the three study groups was interpretation robust to large deviations from the missing data assumptions of the primary analysis (i.e. that those lost to follow‐up were likely to have relapsed) (see Data S8).

### Secondary outcomes

At 3to 4‐years postpartum, none of the differences in self‐reported abstinence between the study groups were statistically significant (using Bonferroni correction). The difference between 12 months incentives and UC approached significance (*P* = 0.019) (see Table 2). Exclusively among those who had been validated as abstinent at 1 year postpartum (*n* = 138), the proportion subsequently validated as abstinent at 3 to 4 years was as follows: UC, 10 of 42 (23.8%); 3 months incentives, 12 of 33 (36.4%); and 12 months incentives, 23 of 63 (36.5%). Table 3 summarises other secondary and exploratory outcomes. Only 5% of participants reported using nicotine replacement therapy, and half reported vaping, with a quarter of these expressing interest in stopping vaping. A quarter reported having a partner who smokes and living with smokers. Approximately a fifth had given birth again since the 12‐month follow‐up and 7% were currently pregnant. Just over half breast fed their baby at the start of the trial, with approximately half of these breastfeeding for at least 4 months. These outcomes were similar across the groups, except rates of vaping and breastfeeding were higher in the incentive groups than UC.

**TABLE 3:** Secondary outcomes by study group among participants followed‐up at 3 to 4 years postpartum.

	Usual care (*n* = 26)	3‐month incentives (*n* = 40)	12‐month incentives (*n* = 57)	Total (*n* = 123)
Using NRT	1/25 (4.0)	4/40 (10.0)	1/56 (1.8)	6/121 (5.0)
Using vapes	10/25 (40.0)	23/40 (57.5)	27/55 (49.1)	60/120 (50.0)
Interested in stopping vaping	2/10 (20.0)	7/23 (30.4)	6/27 (22.2)	15/60 (25.0)
Use of extra cessation support	0/26	0/40	0/57	0/123
Partner smokes	8/26 (30.8)	9/40 (22.5)	14/57 (24.6)	31/123 (25.2)
Living with smokers	8/26 (30.8)	8/40 (20.0)	16/57 (28.1)	32/123 (26.0)
Given birth again since time of 12‐month follow‐up	7/26 (26.9)	7/40 (17.5)	9/57 (15.7)	23/123 (18.7)
Currently pregnant	2/26 (7.7)	3/40 (7.5)	3/57 (5.3)	8/123 (6.5)
Breast fed baby born at start of trial	14/25 (21.9)	20/39 (51.3)	30/55 (46.2)	64/119 (53.8)
Breastfed for ≥4 months	7/25 (28.0)	9/39 (23.1)	15/55 (27.3)	31/119 (26.1)
Mean (SD) time between birth and follow‐up (months)	44.9 (7.3)	43.6 (7.1)	45.1 (8.0)	44.6 (7.6)

*Note*: Data are *n* (%) of participants

Abbreviation: NRT, nicotine replacement therapy.

## DISCUSSION

The findings show that adding 12 months of postpartum incentives to current cessation support for pregnant women in England is likely to maintain long‐term smoking cessation compared with UC. At the 3 to 4‐year follow‐up, offering up to £300 of incentives over 12 months postpartum achieved validated abstinence rates of 16%, compared with rates of 12% when offering £120 over 3 months and 7% for UC. The 9% higher abstinence rate for 12 months incentives vs. UC was statistically significant in the primary outcome analysis, when only adjusting for site, and approached significance in the fully adjusted analysis (*P* = 0.027) using a Bonferroni correction. However, it is important to consider the clinical importance of the observed effect of the intervention as well as the level of statistical significance [22, 23]. In this case, there was a more than twofold increase in abstinence rates in the 12‐month incentives group (OR = 2.44). Combined with the upper CI indicating a possible fivefold to sixfold benefit for the intervention, this suggests an effect that is likely to be clinically meaningful in this context [23]. This conclusion is also supported by the BF, which indicate that for 12 months vs. UC there was good evidence for the alternative hypothesis.

Abstinence rates were 5% higher for 3 months incentives vs. UC, a difference that was not statistically significant. Yet, similar to 12 months incentives, the upper CI suggested a possible fourfold to fivefold benefit for the 3‐month intervention and a point estimate (OR = 1.94), also suggesting an effect that is likely to be clinically relevant. The findings showed a statistically significant linear trend in abstinence across the three groups, from UC to 12‐month incentives, indicating that the longer the duration of the incentives the higher the likelihood of abstinence at 3 to 4‐year follow up. When exclusively examining those who had been previously validated as abstinent at 1 year postpartum, the proportion maintaining validated smoking abstinence at 3 to 4 years was 13% higher in the incentive groups compared with UC. This is consistent with the conclusion for the primary analysis, that financial incentives are likely to support long‐term maintenance of smoking abstinence. One of the keys aims of these interventions is to prevent infants' exposure to second‐hand smoke. The findings suggest that the absolute effect of the interventions weakens over time, however, this is consistent with patterns observed in smoking relapse prevention interventions more generally [24].

### Strengths and limitations

To our knowledge, this is the first study to assess the effectiveness of financial incentives for maintaining postpartum smoking cessation among women who smoked during pregnancy, but ceased by the time their baby was born. Therefore, it is also the first to conduct a long‐term follow‐up of such an intervention. This study has the notable strengths that abstinence was verified biochemically and the follow‐up of 3 to 4 years is sufficiently long to test the sustained benefit of financial incentives.

The study was not powered for such a long‐term follow‐up, however, abstinence rates were higher than anticipated, which increased power, and our primary analysis provides some evidence for an effect, as did both our Bayesian analysis and test for a linear trend. It could still, however, be underpowered, which would explain the lack of statistical significance for the comparison of 12‐month incentives and UC in the fully adjusted analysis (*P* = 0.027). Our conservative approach of using a Bonferroni correction of *P* < 0.017 also contributed to lack of significance. On reflection, *post hoc*, we could have justified including two primary analyses, comparing each incentive intervention with UC using *P* < 0.05 and a secondary analysis comparing the incentive interventions using a Bonferroni correction.

At 3 to 4 years postpartum, the overall follow‐up rate of 63% (i.e. proportion with data for smoking status) (see Figure 1) was modest. Among the two previous trials we identified, which examined interventions to maintain postpartum smoking cessation and conducted a follow‐up beyond 12 months postpartum [14, 15], only one study reported the final follow‐up rate, which at 65% was similar to our follow‐up rate [15]. Follow‐up rates were higher in the incentive groups than UC, consistent with evidence that incentives improve retention [25]. We attempted to reduce the effect of incentives on attrition by offering incentives (i.e. £20 voucher) to all study groups for attending the follow‐up. However, it remains that the differential follow‐up rate between the groups may have affected the results, as missing data were principally analysed as smokers, and this is reflected in the missing data analysis. When handling missing data using complete case analysis or multiple imputation, the difference in abstinence rates between the 12‐month incentive group and UC was no longer significant. Similarly, when using pattern mixture models, the difference between these groups decreased as the missing = smoking assumption was relaxed. The discrepancy between the results for the latter approaches for handling missing data and the approach that assumes that those missing are smoking raises the possibility that there are biases in the observed treatment effect. However, the assumption that data are missing at random is unlikely to apply in this context, (those who relapse typically avoid follow‐up contact while those who succeed are keen to be followed up), and this is a standard approach in smoking cessation trials as advocated by the Russell Standard [17]. Moreover, the characteristics of those who were followed up are similar to those of the total trial sample (see Table 1).

For the primary outcome, only two‐thirds of those reporting abstinence provided biochemical verification. However, this is unlikely to have affected the main findings as the proportion undergoing verification was very similar across the groups, self‐reports of abstinence reflected findings for the primary outcome, and the baseline characteristics of those who did and did not undergo verification were very similar (see Table 2).

We deviated from the Russell Standard [17] and from more recent recommendations [26] in that our primary outcome required reports of lapse‐free abstinence, rather than allowing lapses with up to five cigarettes, and we did not collect data on the number of lapses. Therefore, we could not assess an outcome based on permitting lapses, nor could we include an outcome for 7‐day point‐prevalence abstinence, as recommended [26]. These omitted outcomes are important for detecting delayed quitting. We only followed up participants who achieved abstinence at 1 year, or were not contactable at 1 year, and some participants who had relapsed at 1 year may have subsequently achieved abstinence. If we had included any of the above additional outcomes in secondary analyses it is possible that between‐group differences could have been diluted.

### Comparisons with other studies

Neither of the two previous trials that have examined interventions to maintain postpartum smoking cessation, with follow‐up beyond 12 months postpartum, showed any indication of a benefit of the intervention [14, 15]. One study randomised women in early pregnancy to counselling or UC (*n* = 600) and conducted follow‐ups between 25 and 54 months postpartum [14]. The other study randomised women soon after the birth to counselling or a control group (UC plus self‐help material) (*n* = 304) and followed up participants to 2‐years postpartum [15]. Both studies reported very similar rates of self‐reported sustained abstinence (without biochemical verification) for the two groups.

### Implications for policy and research

The findings provide some evidence for recommending 12 months of postpartum incentives for helping to maintain abstinence for 3 to 4 years postpartum. This evidence should be interpreted with caution as: (i) the *a priori* level of statistical significance was not reached for biochemically verified smoking cessation comparing 12‐month incentives and UC for this long‐term follow‐up; and (ii) unlike financial incentives for smoking cessation during pregnancy [5], generalisability of financial incentives for maintaining postpartum smoking abstinence has not yet been demonstrated. The cost effectiveness of these incentive interventions will be reported elsewhere. This evidence is specific to women in England who, before joining the trial, received incentives and behavioural support to stop smoking during pregnancy. Before the intervention is rolled out, further trials are needed to confirm whether incentives are effective in this population, and if they are also effective in other populations of pregnant and postpartum women (e.g. those who have not ceased smoking with the aid of incentives). There is also a need to assess different schedules and types of financial incentives for smoking cessation during pregnancy and postpartum [27]. Based on existing evidence [5, 6, 7, 8], the English NHS is now supporting a National Smoke‐free Pregnancy Incentives Scheme, such that all pregnant persons who smoke are offered incentives to stop smoking and remain smoke‐free during postpartum [28]. This scheme offers opportunities for further assessments of the impact of financial incentives, both in pregnancy and during postpartum.

## AUTHOR CONTRIBUTIONS

**Michael Ussher:** Conceptualization; investigation; funding acquisition; writing—original draft; methodology; writing—review and editing; formal analysis; project administration; data curation; supervision; resources; validation. **Catherine Best:** Conceptualization; writing—original draft; methodology; writing—review and editing; formal analysis. **Sarah Lewis:** Conceptualization; methodology; writing—review and editing; formal analysis. **Linda Bauld:** Conceptualization; funding acquisition; methodology; writing—review and editing. **Jennifer McKell:** Conceptualization; methodology; writing—review and editing. **Sophie Orton:** Conceptualization; methodology; writing—review and editing. **Sue Cooper:** Conceptualization; methodology; writing—review and editing. **Tim Coleman:** Conceptualization; methodology; writing—review and editing.

## ACKNOWLEDGEMENTS

We acknowledge the support of the senior management team for the Smokefree Pregnancy Programme at NHS Greater Manchester, including Jane Coyne, Andrea Crossfield and Fran Frankland. We thank the specialist maternity treating tobacco dependency teams across NHS Greater Manchester who conducted the follow‐up assessments. Tim Coleman is an NIHR Senior Investigator. The views expressed are those of the author(s) and not necessarily those of the NIHR or the Department of Health and Social Care.

## DECLARATION OF INTERESTS

The funder had no role in considering the study design or in collection, analysis, interpretation of data, writing of the report, or decision to submit for publication. University of Stirling sponsored the study; authors alone decided to publish the paper without influence from the Sponsor. There are no other competing interests to declare.

## CLINICAL TRIAL REGISTRATION

ISRCTN Registry ISRCTN55218215. Date submitted for registration 6 June 2019; date registered 5 July 2019.

## Supporting information


**Data S1.** Supporting Information

**Data S2.** Supporting Information

**Data S3.** Supporting Information

**Data S4.** Supporting Information

**Data S5.** Supporting Information

**Data S6.** Supporting Information

**Data S7.** Supporting Information

**Data S8.** Supporting Information

## Data Availability

The data that support the findings of this study are available on request from the corresponding author. The data are not publicly available due to privacy or ethical restrictions.
