# Supplementary material for: Financial incentives for maintaining postpartum smoking abstinence: 3–4‐year follow‐up of the Financial Incentives for Prevention of Postpartum Return to Smoking (FIPPS) randomised controlled trial
Source: Addiction. 2026 Jul 14;121(10):2725–34. doi: 10.1111/add.70505 (PMC13578832; doi:10.1111/add.70505)
Supplement: Supplementary file 2 [file ADD-121-2725-s007.pdf]

Check for updates

Received: 20 November 2023 | Accepted: 22 February 2024

DOI: 10.1111/add.16487

RESEARCH REPORT

ADDICTION SSA

Effect of 3 months and 12 months of financial incentives on 12-month postpartum smoking cessation maintenance: A randomized controlled trial

Michael Ussher[1,2] | Catherine Best[1] | Sarah Lewis[3] | Jennifer McKell[1] | Tim Coleman[3] | Sue Cooper[3] | Sophie Orton[3] | Linda Bauld[4]

[1]Institute for Social Marketing and Health, University of Stirling, Stirling, UK

[2]Population Health Research Institute, St George's, University of London, London, UK

[3]Faculty of Medicine and Health Sciences, University of Nottingham, Nottingham, UK

[4]Bruce and John Usher Professor of Public Health, Usher Institute and SPECTRUM Consortium, University of Edinburgh, Edinburgh, UK

Correspondence

Michael Ussher, Professor of Behavioural Medicine, Institute of Social Marketing and Health, University of Stirling, Stirling FK9 4LA, UK and Population Health Research Institute, St George's, University of London, Cranmer Terrace, London SW17 0RE, UK.
Email: mussher@sgul.ac.uk

Funding information

Greater Manchester Combined Authority

Abstract

Background and aims: Offering financial incentives is effective for smoking cessation during pregnancy. We tested the effectiveness of financial incentives for maintaining postpartum cessation, comparing 12-month and 3-month incentives with each other and with usual care (UC).

Design, setting and participants: This study was a pragmatic, multi-centre, three-arm randomized controlled trial involving four English, National Health Service, stop smoking services. A total of 462 postpartum women (aged ≥ 16 years) took part, who stopped smoking during pregnancy with financial incentives, validated as abstinent from smoking at end of pregnancy or early postpartum.

Interventions: Interventions comprised (i) UC; (ii) UC plus up to £60 of financial voucher incentives offered to participants and £60 offered to an optional significant-other supporter, over 3 months postpartum, contingent upon validated abstinence ('3-month incentives'); or (iii) UC plus '3-month incentives' plus £180 of vouchers offered to participants over 9 months postpartum, contingent upon abstinence ('12-month incentives').

Measurements: Primary outcome: biochemically validated abstinence at 1 year postpartum. To adjust for testing all comparisons between groups with equal precision, $P < 0.017$ was necessary for significance. Secondary outcomes: self-reported and validated abstinence at 3 months postpartum; self-reported abstinence at 1 year postpartum.

Findings: Primary outcome ascertainment: abstinence was 39.6% (63/159) 12 months incentives, 21.4% (33/154) 3 months incentives and 28.2% (42/149) UC. Adjusted odds ratios [95% confidence interval (CI)] = 12-month versus 3-month incentives OR = 2.41 (95% CI = 1.46–3.96), $P = 0.001$; 12 months versus UC 1.67 (1.04–2.70), $P = 0.035$; 3 months versus UC 0.69 (0.41–1.17), $P = 0.174$. Bayes factors indicated that for 12-month versus 3-month incentives and 12 months versus UC there was good evidence for the alternative hypothesis, and for 3 months versus UC there was good evidence for the null hypothesis.

Conclusions: This randomized controlled trial provides weak evidence that up to £300 of voucher incentives over 12 months is effective for maintaining smoking abstinence

This is an open access article under the terms of the Creative Commons Attribution License, which permits use, distribution and reproduction in any medium, provided the original work is properly cited.
© 2024 The Authors. *Addiction* published by John Wiley & Sons Ltd on behalf of Society for the Study of Addiction.

postpartum compared with usual care. There was good evidence that 12-month incentives are superior to those over only 3 months, for which there was no evidence of effectiveness relative to usual care.

KEYWORDS

Abstinence, financial incentives, intervention, postpartum, pregnancy, randomized controlled trial, relapse, smoking, vouchers

INTRODUCTION

More women stop smoking during pregnancy than at any other time; approximately half are likely to cease smoking 'spontaneously' [1]. This is an opportunity to help women stop smoking permanently. Most women who cease smoking in pregnancy say they wish to remain abstinent [2]. However, up to three-quarters are likely to return to smoking within 6 months of giving birth [3], increasing their risks of smoking-related illness and mortality [4, 5] as well as their children's risks of passive smoking [6] and of becoming smokers [7]. Also, there are marked health inequalities, as women with lower socio-economic status and education are more likely to relapse [8]. Reducing postpartum return to smoking may be one of the few interventions that can reduce health inequalities in early life. A review of 15 trials assessing interventions for reducing postpartum return to smoking, focusing on education and counselling, found no significant benefit of the interventions [9]. New approaches need to be developed and evaluated.

In 2023, a review of 12 trials showed that offering financial incentives is highly effective for smoking cessation during pregnancy, with those offered incentives twice as likely to remain abstinent from smoking compared with those not offered incentives [10]. In the United Kingdom, since 2021 the National Institute of Health and Care Excellence has recommended financial incentives for pregnant women who smoke, based on their economic modelling demonstrating cost-effectiveness [11]. Such interventions are now being implemented in routine prenatal care [12]. Prompted by recent demonstrations of cost-effectiveness in UK and US trials [13, 14] and further evidence of effectiveness in a UK trial [15], the UK government announced that all pregnant women in England who smoke will be offered incentives to stop smoking by the end of 2024 [16].

It is plausible that extending the offer of incentives into the postpartum period will assist maintenance of smoking cessation. The above review [10] identified five US studies that offered incentives during postpartum [17–21], with incentives ranging from \$100 for postpartum women and \$50 for a significant-other supporter (SOS) over 2 months [17] to \$520 for postpartum women over 3 months [21]. These incentives appeared to be acceptable to participants. However, women who smoked were randomized in early pregnancy to examine the impact of incentives on smoking cessation during pregnancy; therefore, it was not possible to examine the separate effect of postpartum incentives on maintenance of postpartum smoking cessation among those achieving abstinence at end-of-pregnancy. Moreover, the number of women abstinent at end-of-pregnancy was too small (range = 19–41 women) to examine trends among those offered incentives versus those not offered. This paper reports a large randomized controlled trial which is the first, to our knowledge, to test whether postpartum financial incentives can aid maintenance of postpartum smoking cessation. Specific hypotheses were that: (i) 12-months and 3-months incentives will be more effective than usual care (UC); (ii) 12-months incentives will be more effective than 3-months incentives; and (iii) there will be a significant linear trend in abstinence across the three study groups, with rates increasing from UC to 12-months incentives.

METHODS

Design

The FIPPS study (Financial Incentives for Prevention of Postpartum return to Smoking [22]) was a pragmatic, multi-centre, phase III, parallel-group, three-arm, individually randomized controlled trial. It compared smoking abstinence rates at 3 months and 1 year postpartum for three groups: (i) UC, (ii) UC plus financial incentives offered for up to 3 months postpartum, and (iii) UC plus incentives offered for 12 months post-partum among women who were abstinent from smoking at end-of-pregnancy.

Participants

Eligible women were participating in a programme offering financial incentives for smoking cessation during pregnancy (see published protocol/Supporting information, Data S1: A [22]), between 34 weeks gestation and 2 weeks postpartum, self-reported not smoking a single puff of a cigarette for at least 4 weeks, exhaled carbon monoxide (CO) reading was < 4 parts per million (p.p.m.), aged 16 years or older, intended remaining abstinent from smoking after the birth, English speakers and willing and able to give written informed consent for participation. In order to take their own CO measurements, they required a mobile phone compatible with the iCO™ (single-person use) CO monitor (Bedfont Scientific Ltd, Maidstone, UK [23]) application. Recruitment was from National Health Service stop smoking services (midwife-led and non-midwife-led) serving four maternity hospitals in Greater Manchester, UK, covering large areas of deprivation and including a city, several provincial towns, suburban and rural areas. Births at the sites ranged from 2230 to 12 150 per year.

13600443, 2024, 8, Downloaded from https://onlinelibrary.wiley.com/doi/10.1111/add.16487 by Test, Wiley Online Library on [17/10/2024]. See the Terms and Conditions (https://onlinelibrary.wiley.com/terms-and-conditions) on Wiley Online Library for rules of use; OA articles are governed by the applicable Creative Commons License

Interventions

During postpartum, control participants received care as usual, with no support for avoiding return to smoking. During pregnancy all participants, as part of routine care, received brief, face-to-face, individual advice regarding maintaining smoking abstinence during both postpartum and long-term. All participants were offered a £20 voucher for completing research assessments at 3 months and 1 year postpartum.

All participants in the two individually delivered intervention groups received UC and were also offered financial incentives for up to either 3 or 12 months postpartum by experienced, trained stop smoking advisers. They could also identify a SOS to help them to remain abstinent, who was also offered incentives. Incentives were Love2shop shopping vouchers, given or posted. For participants, payments were conditional on self-report of not smoking a single puff of a cigarette since their last quit date during pregnancy and an exhaled CO reading of < 8 p.p.m. During pregnancy, due to metabolic and respiratory changes, a CO cut-off of < 4 p.p.m. is recommended [24, 25]; out of pregnancy a cut-off of < 8 p.p.m. is more standard [26]. For the SOS, the payment was conditional on an exhaled CO reading of < 8 p.p.m., irrespective of whether they had been a smoker. Initially, interventions were delivered face-to-face at a hospital facility. During COVID-19 restrictions, most participants opted for telephone contact (using iCO monitor) or a home visit, and the SOS was confirmed as abstinent based on self-report alone (see protocol for COVID-19 adaptations [22]). Interventions sessions lasted for approximately 7 minutes.

In the 3-months incentive group participants were offered up to £60 of incentives, with £20 offered at 1, 2 and 3 months postpartum. At 3 months postpartum, SOSs were offered a £60 voucher if both participant and SOS were validated as abstinent.

In the 12 months incentive group, in addition to the incentives offered to participants and SOSs in the 3 months group, participants were offered £60 at 6, 9 and 12 months postpartum. In total, this group was offered £300. All the interventions were delivered uniformly across the sites.

Procedures

On joining the pregnancy stop smoking programme women were informed that, if they were abstinent at the end of their pregnancy, they may be invited to join a study examining the effects of offering shopping vouchers on abstinence during the first year after their baby's birth. Those reporting not currently smoking and confirmed as abstinent (CO < 4 p.p.m.) by the stop smoking service at approximately 32 weeks gestation were given a 'generic' participant information sheet. Stop smoking advisers enrolled participants at between 34 weeks gestation and 4 weeks postpartum. Baseline and consent questions were completed before the adviser requested automated randomized group allocation on-line, ensuring concealment, with the participant present. Participants were randomized to one of three conditions within site. During COVID-19 restrictions, when written consent for trial participation could not be obtained face-to-face, written or verbal consent was obtained by 'distanced' methods (see protocol [22]).

Randomization (1:1:1 allocation) was stratified by site, using randomly permuted blocks of varying size. The randomization sequence was computer-generated and stored in a secure online program. Due to the nature of the intervention, in this pragmatic trial, it was not possible to blind participants to treatment allocation, nor were the advisers conducting the assessments blinded to allocation, as the advisers both delivered the intervention and conducted assessments, with the assessments being part of the intervention. The statisticians were blinded to allocation.

The trial protocol was approved by the North-West-Liverpool Central Research Ethics Committee (ref: 18/NW/0838). Trained researchers at the University of Stirling added data to a secure trial database and conducted data monitoring (see Supporting information, Data S2: B). Trial planning, including preparation of participant materials, included two patient and public involvement and engagement (PPIE) representatives who had smoked during pregnancy. We carefully assessed the burden of the trial interventions on participants. There was additional PPIE representation on the Trial Steering Committee that included input on plans for dissemination of the findings.

Measures

At 3 months and 1 year postpartum follow-ups, stop smoking service advisers conducted assessments and were trained to do so. Initially, advisers assessed participants' smoking status over the telephone (up to five contact attempts). Those reporting not smoking a single puff (since at least 4 weeks prior to randomization) were asked to biochemically verify their smoking status with their adviser at either a face-to-face appointment or remotely during COVID-19 restrictions, as outlined in the protocol [22].

The primary outcome was self-report of sustained, lapse-free, smoking abstinence at 1 year postpartum, biochemically validated by an exhaled CO reading of < 8 p.p.m., and/or saliva cotinine or anabasine estimation. Women were verified as not smoking if their saliva cotinine concentration was < 10 ng/ml [27], or where current nicotine replacement therapy or e-cigarette use was reported, saliva anabasine was ≤ 0.2 ng/ml [28]. Where possible, both a CO reading and saliva sample confirmed abstinence, where only one of these measures was collected that was used to confirm abstinence. At 3 months postpartum, if a participant could not be contacted or if they self-reported abstinence but this was not validated by a CO reading (due mainly to COVID restrictions), they could still satisfy the primary outcome if they were validated as abstinent at 1 year postpartum. If at 3 months they reported smoking or had a CO reading ≥ 8 p.p.m. they were counted as having relapsed for the primary outcome.

Secondary assessments, at 3 months and 1 year postpartum, were the proportion of women self-reporting: abstinence from

13600443, 2024, 8, Downloaded from https://onlinelibrary.wiley.com/doi/10.1111/add.16487 by Test, Wiley Online Library on [17/10/2024]. See the Terms and Conditions (https://onlinelibrary.wiley.com/terms-and-conditions) on Wiley Online Library for rules of use; OA articles are governed by the applicable Creative Commons License

smoking; use of nicotine replacement therapy, electronic cigarettes and heat-not-burn products; a partner who smokes; smokers living in their home; use of additional smoking cessation support beyond that in the trial; and use of the iCO monitor beyond that required for assessments. We also assessed biochemically validated (CO < 8 p.p.m.) self-reported abstinence from smoking up to 3 months postpartum. Assessments for cost-effectiveness and process evaluation are reported in the protocol [22] and the findings will be reported elsewhere.

Data analysis

Analyses followed a pre-specified statistical analysis plan (https://osf.io/nckj9/https://osf.io/nckj9/, registered 7 June 2022) using Stata (StataCorp, release 17; College Station, TX, USA) and SPSS version 29 (Chicago, IL, USA). Hypothesis tests were two-sided. The intention-to-treat population was defined as 'all participants who were randomly assigned to the study and eligible to participate'. The planned sample size was 900 participants (300 per trial group), giving 90% power at 1.7% significance, to detect a difference in abstinence rates across any two groups of 13.6%. The estimate of 13.6% difference was based on the difference found in a UK study of pregnancy incentives [29]. With the sample size achieved (n = 462), and the above estimate of effect sizes, we had 0.6 power for 3-month versus 12-month incentives and 0.82 and near 1.0 power for no incentives versus 3-month and 12-month incentives, respectively. For examining a linear contrast across the three groups we had almost 1.0 power.

Baseline data were summarized descriptively by study group for all participants, and for participants who provided smoking status for the primary outcome [30]. We used χ^2 tests to compare follow-up rates between study groups. For analysis of biochemically validated smoking outcomes, where outcomes were missing participants were assumed to be smokers [26].

Analysis for the primary outcome used a mixed-effects logistic regression model with randomized treatment group as a fixed effect, and recruiting site adjusted for as a random effect (random intercept only, to control for non-independence of observations within sites) [31], with pairwise comparisons between treatment groups, using a significance level of $P < 0.017$ (Bonferroni correction for multiple comparisons [32]). Bayes factors (BFs) were produced using an on-line calculator (http://www.lifesci.sussex.ac.uk/home/Zoltan_Dienes/inference/Bayes.htm) to examine whether there was evidence for the alternative (H1) or null (H0) hypothesis. Usual conventions were applied (i.e. good evidence for H1 over H0 if BF > 3; good evidence for H0 over H1 if BF < 1/3; otherwise, inconclusive evidence). We set the hypothesized odds ratio (OR) to 1.5, but also examined the effect of varying the lower bound from 1.2 to 2, using a one-tailed test. We also looked for a linear trend in abstinence across the three groups, from control to 12-month incentives (significance $P < 0.05$).

We conducted secondary analyses for the primary outcome, adjusting for key baseline variables predicted to be related to postpartum smoking status [8, 33] (i.e. education, A-level or equivalent or higher versus lower qualifications), cigarette consumption before pregnancy, depression (Edinburgh Depression Scale score) [34] and age. We also conducted a sensitivity analysis to examine effects on the primary outcome for other baseline variables that had a marked difference between groups and were associated with smoking status. We compared rates of validation of smoking abstinence and sizes of effect of interventions between the pre- and post-COVID-19 periods (cut-off 16 March 2020) in sensitivity analyses to assess the impact of COVID on the primary analysis. For the primary outcome, sensitivity to missing data was assessed using three methods: complete case analysis, multiple imputation by chained equations and a pattern-mixture model to assess sensitivity to deviations from the missing data assumptions of the primary analysis.

Further secondary analysis, with the same adjustments as for the primary outcome, included group comparisons for self-reported smoking cessation at 3 months and 1 year postpartum and validated smoking cessation at 3 months postpartum. Descriptive statistics were generated for 3 months and 1 year postpartum assessments of use of nicotine products, smoking in the home, partner smoking status, use of additional smoking cessation support and use of the iCO monitor beyond that required for research assessments. The number/percentage of SOSs who received a £60 incentive was reported by study group. We added some outcomes that were not pre-specified: we presented the proportion in the 12-month incentives group verified as abstinent at 6 months and 9 months postpartum to show the progression of relapse across time; we reported the proportion counted as having returned to smoking by the first month postpartum, as this was notably high. No outcome data were excluded.

FINDINGS

From 22 March 2019 to 31 August 2021, 661 women were screened, 180 of whom (27.2%) were ineligible; 481 (72.8%) participants were randomized (Figure 1). Subsequently, 18 participants were identified as being ineligible (due mainly to baseline CO reading > 3 p.p.m., n = nine). The independent trial steering committee reviewed each case (blinded to study group) and recommended withdrawing all 18 participants [35]. A further participant was withdrawn due to the infant dying. Data for the remaining 462 individuals were analysed (12-month incentives n = 159, 3-month incentives n = 154, UC n = 149). Due to the interruptions of COVID-19, including lack of face-to-face screening of women's smoking status during pregnancy [36] and reduced staffing due to illness or 'shielding', trial recruitment did not meet the target of 900 participants randomized, despite an extended period of recruitment. We did not have the resources to further extend recruitment.

13600443, 2024, 8, Downloaded from https://onlinelibrary.wiley.com/doi/10.1111/add.16487 by Test, Wiley Online Library on [17/10/2024]. See the Terms and Conditions (https://onlinelibrary.wiley.com/terms-and-conditions) on Wiley Online Library for rules of use; OA articles are governed by the applicable Creative Commons License

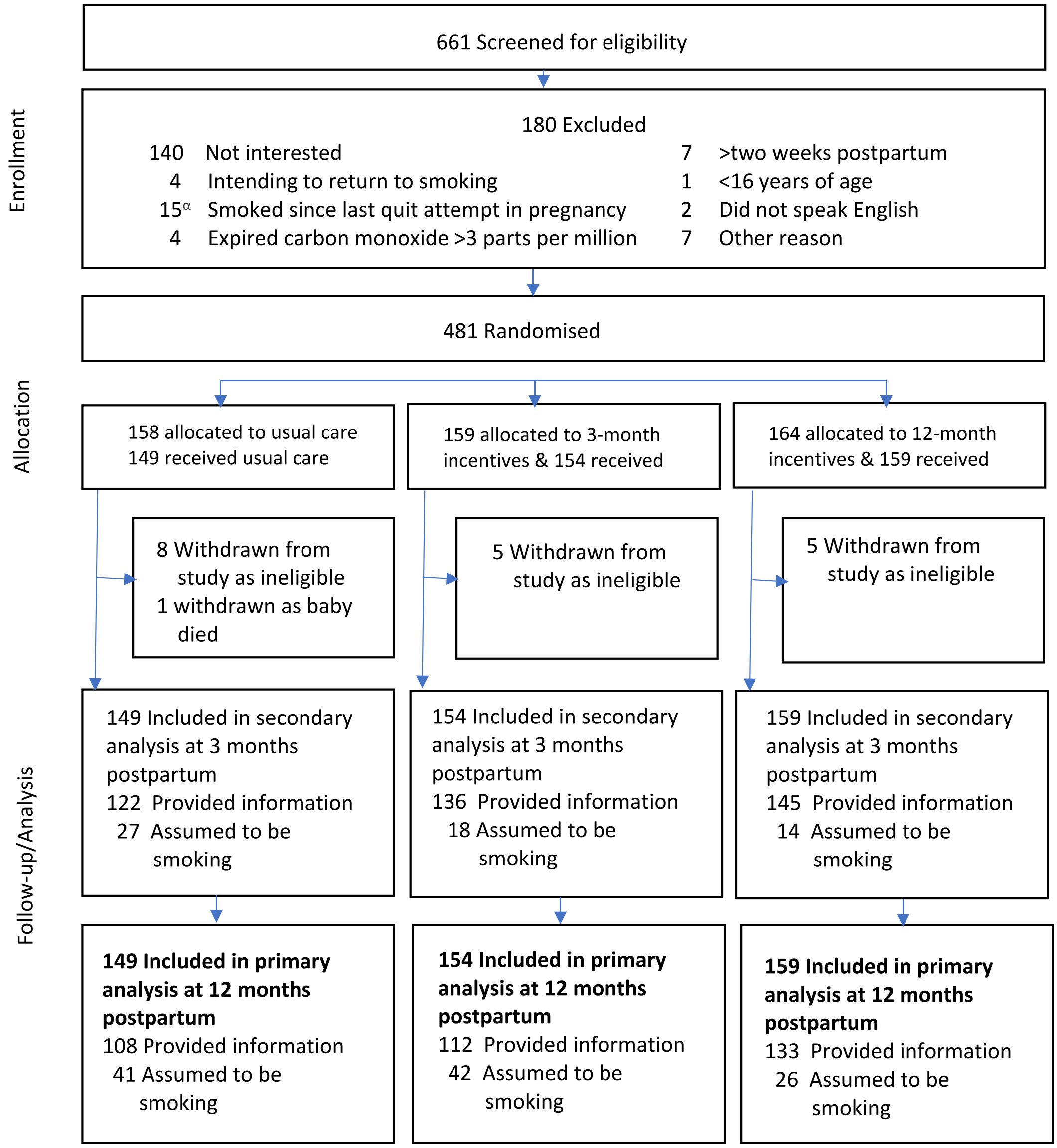

FIGURE 1 Trial profile of potential participants, participants who were enrolled and randomly assigned to a group and participants whose data were analysed. $^{\alpha}$ eligibility criterion was subsequently replaced with 'self-reported not smoking a single puff of a cigarette for at least 4 weeks', to include those who had had some earlier lapses but were abstinent ≥ 4 weeks. Those recruited before this amendment were all abstinent for ≥ 4 weeks, and following this amendment no one was excluded for having smoked in the last 4 weeks.

Baseline data

The mean age of the 462 participants was 28.3 years; 87.2% were recruited in late pregnancy (mean gestation 36.4 weeks) and 12.8% joined in early postpartum (Table 1). In 95.5% of cases the intervention was delivered by a midwife-led stop smoking service; therefore, we did not adjust the analysis for type of service. At baseline, use of nicotine replacement therapy was reported by 13.2% of participants and e-cigarette use by 28.6%. On average, participants reported being abstinent from smoking for 22.7 weeks. The groups had similar baseline characteristics, except that the 12-month incentives group tended to report higher levels of education and was more likely to have a SOS than the other groups, the 12-month and UC groups reported more confidence for maintaining smoking abstinence than the 3-month group, and the 3- and 12-month groups reported more use of nicotine replacement therapy and e-cigarettes than UC.

Primary outcome

Table 2 presents the primary outcome analysis. Follow-ups were completed on 18 October 2022. Overall, 76.4% (353/462) of participants completed self-report of smoking status at 1 year postpartum, with rates of completion approximately 11% higher for 12-month incentives than the other two groups (P = 0.029). Baseline characteristics of those who did and did not self-report smoking status were similar (Table 1). There were some differences in characteristics of those who

13600443, 2024, 8, Downloaded from https://onlinelibrary.wiley.com/doi/10.1111/add.16487 by Test, Wiley Online Library on [17/10/2024]. See the Terms and Conditions (https://onlinelibrary.wiley.com/terms-and-conditions) on Wiley Online Library for rules of use; OA articles are governed by the applicable Creative Commons License

TABLE 1 Baseline characteristics of all participants in analysis and participants providing smoking status for primary outcome, by study group. Data are number (%) of participants unless otherwise stated.

	All participants in analysis				Participants who provided smoking status for primary outcome		
	Usual care (*n* = 149)	3-month incentives (*n* = 154)	12-month incentives (*n* = 159)	Total (*n* = 462)	Usual care (*n* = 108)	3-month incentives (*n* = 112)	12-month incentives (*n* = 133)
Demographic characteristics							
Maternal age in years, mean (SD)	27.7 (5.6)	28.6 (5.4)	28.6 (5.9)	28.3 (5.7)	27.6 (5.5)	28.8 (5.4)	28.7 (6.0)
A-level, degree or equivalent	72 (48.3)m[2]	77 (50.0)	93 (58.5)	242 (52.4)m[2]	72 (50.9)m[2]	56 (50.0)	81 (60.9)
White ethnicity	140 (94.0)	142 (92.2)	145 (91.2)	427 (92.4)	103 (95.4)	105 (93.8)	121 (91.0)
Type of stop smoking service							
Midwife-led	144 (96.6)	145 (94.2)	152 (95.6)	441 (95.5)	106 (98.1)	107 (95.5)	127 (95.5)
Smoking history							
Pre-pregnancy cigarettes smoked a day, mean (SD)	12.9 (7.2)m[1]	13.5 (6.7)	12.4 (6.8)m[2]	12.9 (6.9)m[3]	13.2 (7.0)m[1]	14.0 (6.9)	12.5 (6.9)m[2]
Weeks of continuous smoking abstinence, mean (SD)	22.8 (6.4)	22.1 (6.6) m[1]	23.3 (6.3) m[1]	22.7 (6.5)m[2]	22.8 (6.3)	21.8 (6.8) m[1]	23.4 (6.4) m[1]
Expired CO level p.p.m., mean (SD)	1.1 (0.8)	1.2 (0.7)m[1]a	1.2 (0.8)m[1]b	1.2 (0.8)m[2]	1.1 (0.8)	1.2 (0.7)m[1]a	1.3 (0.8)m[1]b
Very or extremely confident in maintaining smoking abstinence	107 (71.8)m[1]	99 (64.3)	110 (69.2)	316 (68.4)m[1]	72 (66.7)m[1]	72 (64.3)	89 (66.9)
Uses nicotine replacement therapy	15 (10.1)	22 (14.3)	24 (15.1)	61 (13.2)	13 (12.0)	15 (13.4)	21 (15.8)
Uses electronic cigarettes	36 (24.2)	51 (33.1)	45 (28.3)	132 (28.6)	25 (23.1)	34 (30.4)	42 (31.6)
Uses heat-not-burn	4 (2.7)	2 (1.3)	4 (2.5)	10 (2.2)	4 (3.7)	1 (0.9)	4 (3.0)
Use of smoking cessation support beyond that in trial	0	0	0	0		0	0
Partner smokes	47 (31.5)	49 (31.8)	49 (30.8)	145 (31.4)	34 (31.5)	36 (32.1)	40 (30.1)
Living with smokers	49 (32.9)	56 (36.4)	57 (35.8)	162 (35.1)	34 (31.5)	42 (37.5)	48 (36.1)
Has significant-other supporter	66 (44.3)	72 (46.8)	86 (54.1)	224 (48.5)	49 (45.4)	53 (47.3)	72 (54.1)
Pregnancy							
Weeks pregnant, mean (SD)[c]	36.4 (1.0)	36.3 (1.0)	36.5 (1.2)	36.4 (1.1)	36.4 (1.1)	36.3 (1.0)	36.5 (1.1)
Days postpartum, mean (SD)[d]	8.1 (5.2)	8.4 (4.1)	7.6 (4.1)	8.0 (4.4)	7.7 (5.8)	8.8 (4.7)	7.3 (4.4)
Parity, mean (SD)	1 (1.1)m[2]	1.2 (1.5)	1.0 (1.1)	1.1(1.2)m[2]	0.9 (1.1)m[1]	1.2 (1.6)	1.0 (1.1)
Intend to bottle and breastfeed	25 (16.8)	23 (14.9)	30 (18.9)	78 (16.9)	16 (14.8)	17 (15.2)	25 (18.8)
Intend to breastfeed only	77 (51.7)	75 (48.7)	78 (49.1)	230 (49.8)	58 (53.7)	51 (45.5)	69 (51.9)

(Continues)

13600443, 2024, 8, Downloaded from https://onlinelibrary.wiley.com/doi/10.1111/add.16487 by Test, Wiley Online Library on [17/10/2024]. See the Terms and Conditions (https://onlinelibrary.wiley.com/terms-and-conditions) on Wiley Online Library for rules of use; OA articles are governed by the applicable Creative Commons License

TABLE 1 (Continued)

	All participants in analysis				Participants who provided smoking status for primary outcome		
	Usual care (*n* = 149)	3-month incentives (*n* = 154)	12-month incentives (*n* = 159)	Total (*n* = 462)	Usual care (*n* = 108)	3-month incentives (*n* = 112)	12-month incentives (*n* = 133)
Intend to breastfeed for ≥ 4 months	54 (36.2)	48 (31.2)	48 (30.2)	150 (32.5)	40 (37.0)	35 (31.3)	43 (32.3)
Alcohol use disorders identification, consumption							
Risk drinking (score ≥ 5)	2 (1.3)	4 (2.6)	3 (1.9)	9 (1.9)	1 (0.9)	1 (20.9)	3 (2.3)
Edinburgh Depression Scale (EDS)							
Overall EDS scores, mean (SD)	6.0 (5.0)m[4]	5.4 (5.1)m[5]	5.3 (4.7)m[2]	5.6 (4.9)m[11]	6.0 (5.0)m[4]	5.4 (5.1)m[5]	5.3 (4.7)m[2]
Major depressive disorder (EDS ≥ 11)	29 (19.5)m[4]	29 (18.8)m[5]	27 (17.0)m[2]	85 (18.4)m[11]	22 (20.4)m[4]	20 (17.9)m[5]	23 (17.3)m[2]

m[ ] Missing data [number missing]; SD = standard deviation; CO = carbon monoxide; p.p.m. = parts per million.
[a]Missing due to medical condition;
[b]missing due to COVID-19 distancing;
[c]relates to those recruited in pregnancy;
[d]relates to those recruited postpartum.

did and did not undergo biochemical verification of abstinence (see Supporting information, Data S3: C). Overall, among those reporting smoking abstinence, 75.3% underwent verification and rates of providing this verification were approximately 16% lower in the 3-month incentive group compared with the other groups (*P* = 0.063).

For the primary analysis, adjusting only for site, validated abstinence was higher for 12-month incentives (39.6%) compared with 3-month incentives (21.4%); adjusted odds ratio (aOR) 2.41, 95% CI = 1.46 to 3.96; percentage difference 18%, 95% CI = 8–28%), *P* = 0.001. The difference in validated abstinence between 12-month incentives and UC (28.2%) was not significant with the Bonferroni correction; aOR = 1.67, 95% CI = 1.04–2.70; percentage difference 11%, 95% CI = 1–22% (*P* = 0.035). Nor was the difference significant between 3-month incentives and UC (aOR = 0.69, 95% CI = 0.41–1.17); percentage difference 7% (95% CI = 16% lower to 3% higher), *P* = 0.174). We made the a priori decision to fit a model with centre as a random effect [31]. Despite the degree of clustering being negligible we retained this model, as it was pre-specified. In an alternative model specification including centre as a fixed effect there was no impact on study findings, nor was there a site × treatment interaction.

BFs indicated that for 12-month versus 3-month incentives, and for 12 months versus UC, there was good evidence for H1 (BF > 3). At a hypothesized effect size of 1.5, the BF for 3 months versus UC was 0.27, implying good evidence for H0, although inconclusive for an expected effect size of 1.37 or less. There was a significant linear trend in the proportion abstinent between UC, 3-month and 12-month incentives (*P* = 0.025), although ORs suggested a non-linear association. When examining the effects of pre- versus post-COVID periods on the primary outcome, and on rates of validation of self-reports, there were no apparent differences. Strenuous efforts were made to maintain study rigour, despite the disruption of COVID.

In a fully adjusted model, when further adjusting for pre-defined baseline maternal variables that were predicted to be related to smoking status, and for baseline variables that had a marked difference between groups and were associated with smoking status (i.e. whether support was provided by a SOS, intention to breastfeed, living with smokers, partner smokes), primary outcome findings were similar: validated smoking abstinence was higher for 12-month incentives compared with 3-month incentives (aOR = 2.25, 95% CI = 1.35–3.77, *P* = 0.002). The difference in validated abstinence between 12-month incentives and UC was not significant (aOR = 1.56, 95% CI = 0.95–2.57; *P* = 0.082), nor was there a significant difference between 3-month incentives and UC (aOR = 0.69, 95% CI = 0.40–1.19; *P* = 0.183).

The findings did not change in the complete case analysis (12-month versus 3-month incentives: aOR = 2.15, 95% CI = 1.27–3.66, *P* = 0.005; 12-month incentives versus UC: aOR = 1.41, 95% CI = 0.84–2.37, *P* = 0.187; 3-month incentives versus UC: aOR = 0.66, 95% CI = 0.37–1.15, *P* = 0.141) or by multiple imputation of chained equations (12-month versus 3-month incentives: aOR = 1.95, 95% CI = 1.16–3.29, *P* = 0.012; 12-month incentives versus UC: aOR = 1.32, 95% CI = 0.79–2.20, *P* = 0.274; 3-month incentives versus UC: aOR = 0.68, 95% CI = 0.39–1.20, *P* = 0.179).

13600443, 2024, 8, Downloaded from https://onlinelibrary.wiley.com/doi/10.1111/add.16487 by Test, Wiley Online Library on [17/10/2024]. See the Terms and Conditions (https://onlinelibrary.wiley.com/terms-and-conditions) on Wiley Online Library for rules of use; OA articles are governed by the applicable Creative Commons License

13600443, 2024, 8, Downloaded from https://onlinelibrary.wiley.com/doi/10.1111/add.16487 by Test, Wiley Online Library on [17/10/2024]. See the Terms and Conditions (https://onlinelibrary.wiley.com/terms-and-conditions) on Wiley Online Library for rules of use; OA articles are governed by the applicable Creative Commons License

TABLE 2 Primary outcome derivation and primary analysis by study group.

At 1-year postpartum	Usual care (*n* = 149)	3-month incentives (*n* = 154)	12-month incentives (*n* = 159)	Total (*n* = 462)
Self-reported smoking status[†]				
Abstinent	53/149 (35.6)	53/154 (34.4)	81/159 (50.9)	187/462 (40.5)
Smoking	55/149 (36.9)	59/154 (38.3)	52/159 (32.7)	166/462 (35.9)
Missing self-report due to no contact	41/149 (27.5)	42/154 (27.3)	26/159 (16.4)	109/462 (23.6)
Self-reported as abstinent and underwent biochemical verification test				
Yes	45/56 (80.4)	35/55 (63.6)	69/87 (79.3)	149/198 (75.3)
No[a]	11/56 (19.6)	20/55 (36.4)	18/87 (20.7)	49/198 (24.7)
Verification test changed outcome from abstinent to smoking (i.e. failed test)				
Yes	3/45 (6.7)	2/35 (5.7)	6/69 (8.7)	11/149 (7.4)
No	42/45 (93.3)	33/35 (94.3)	63/69 (91.3)	138/149 (92.6)
Biochemically verified smoking status (primary analysis)				
Abstinent	42[b]/149 (28.2)	33[c]/154 (21.4)	63[d]/159 (39.6)	138/462 (29.9)
Smoking	107/149 (71.8)	121/154 (78.6)	96/159 (60.4)	324/462 (70.1)

Data are number (%) of participants.
[a]In five cases lack of a verification test was due to insufficient saliva to conduct the analysis (control = 1, 3-month incentives = 2, 12-month incentives = 3).
[b]Five participants had self-report only at 3-month follow-up and three could not be followed-up at 3 months.
[c]Four participants had self-report only at 3-month follow-up.
[d]10 participants had self-report only at 3-month follow-up.
[†]Group comparisons for self-reported abstinence, fully adjusted odds ratios (95% confidence intervals: 12-month versus 3-month incentives 1.90 (1.19−3.03), *P* = 0.008; 12-month incentives versus usual care 1.88 (1.17–3.03), *P* = 0.009; 3-month incentives versus usual care 0.99 (0.61–1.62), *P* = 0.982. Excludes 11 individuals who self-reported abstinence but failed verification test.

Pattern-mixture modelling showed that for the comparison of 12-month and 3-month incentives interpretation was robust to large deviations from the missing data assumptions of the primary analysis. For comparison of UC with the two incentive groups, the interpretation was less robust (Supporting information, see Data S4: D).

Secondary outcomes

At 1 year postpartum, a significant difference was observed in self-reported abstinence for 12-month incentives compared with both 3-month incentives and UC, but not between 3-month incentives and UC (see Table 2). Other secondary and exploratory outcomes are summarized in Table 3. At 3 months postpartum, overall, 87.2% (403/462) of participants provided a self-report of smoking status, with rates higher in the incentive groups than UC ($P = 0.044$). Among those reporting smoking abstinence, 75.2% underwent biochemical verification, with similar rates among study groups ($P = 0.714$). At 3 months postpartum, there were no significant group differences for either self-reported or validated abstinence. The percentage of SOSs who received a £60 incentive (at 3 months postpartum) was similar in the two incentive groups. Two participants reported returning to smoking before the birth (3-month incentive group = 1, 12-month incentive group = 1). Initial relapse rates were high, with 41% (188/462) counted as returning to smoking in the first month postpartum [UC = 51% (76/149), 3-month incentives 35% (54/154), 12-month incentives 37% (58/159)]. In the 12-month incentives group, participants progressively returned to smoking between the 3-month and 1-year assessments; at 6 and 9 months postpartum 53% (85/159) and 46% (73/159), respectively, were validated as abstinent. At both 3-month and 1-year follow-ups, fewer than 8% of participants reported using nicotine replacement therapy, and more than a quarter reported using electronic cigarettes, having a partner who smokes and living with smokers.

DISCUSSION

To our knowledge, this is the first study to examine the effectiveness of postpartum financial incentives to aid postpartum smoking cessation among women who smoked during pregnancy but had quit by the time their baby was born. It shows substantial interest in the intervention and that adding a 12-month programme of postpartum incentives to current cessation support for pregnant women in England can help to maintain smoking cessation compared with a 3-month programme. Offering up to £300 of incentives over 12 months postpartum achieved validated abstinence rates of 40%. This compared with rates of 21% when offering £120 over 3 months and rates of 28% for UC.

TABLE 3 Secondary outcomes by study group. Data are number (%) of participants.

	Usual care (*n* = 149)	3-month incentives (*n* = 154)	12-month incentives (*n* = 159)	Total (*n* = 462)
Self-reported smoking status at 3 months postpartum†				
Abstinent	100/149 (67.1)	104/154 (67.5)	123/159 (77.4)	327/462 (70.8)
Smoking	22/149 (14.8)	32/154 (20.8)	22/159 (13.8)	76/462 (16.5)
Missing self-report due to no contact	27/149 (18.1)	18/154 (11.7)	14/159 (8.8)	59/462 (12.8)
Self-reported as abstinent at 3 months postpartum and underwent verification test[a]				
Yes	73/100 (73.0)	81/104 (77.9)	92/123 (74.8)	246/327 (75.2)
No	27/100 (27.0)	23/104 (12.1)	31/123 (25.2)	81/327 (24.8)
Biochemical verification at 3 months postpartum††				
Abstinent	73/149 (49.0)	81/154 (52.6)	92/159(57.9)	246/462 (53.3)
Smoking	76/149 (51.0)	73/154 (57.4)	67/159 (42.1)	216/462 (46.8)
Uses NRT				
At 3 months postpartum	10/118 (8.5)	8/128 (6.3)	12/135 (8.9)	30/381 (7.9)
At 1 year postpartum	2/78 (2.6)	4/74 (5.4)	3/102 (2.9)	9/254 (3.5)
Uses electronic-cigarettes				
At 3 months postpartum	25/118 (21.2)	36/128 (28.1)	39/135 (28.9)	100/381 (26.2)
At 1 year postpartum	20/78 (25.6)	30/74 (40.5)	31/102 (30.4)	81/254 (31.9)
Uses heat-not-burn				
At 3-months postpartum	0/118	1/128 (0.8)	1/135 (0.7)	2/381 (0.5)
At 1 year postpartum	0/78	0/74	0/102	0/254
Additional use of iCO monitor for > 6 days				
At 3 months postpartum	1/73 (1.4)	7/83 (8.4)	2/8 (0.7)	10/236 (4.2)
At 1 year postpartum	0/54	2/52 (3.8)	3/60 (5.0)	5/166 (3.0)
Use of extra cessation support				
At 3 months postpartum	1/118 (0.8)	0/128	0/135	1/381 (0.3)
At 1 year postpartum	0/78	0/74	0/102	0/254
Partner smokes				
At 3 months postpartum	26/118 (22.0)	36/128 (28.1)	39/135 (24.4)	97/379 (25.6)
At 1 year postpartum	28/78 (35.9)	23/74 (31.1)	21/101 (20.8)	72/254 (28.4)
Living with smokers				
At 3 months postpartum	31/118 (26.3)	38/127 (29.9)	36/135 (26.7)	105/380 (27.6)
At 1 year postpartum	30/78 (38.5)	23/74 (31.1)	19/102 (18.6)	72/254 (28.4)
SOS received incentive	NA	33/72 (45.8)	38/86 (44.2)	71/158 (44.9)

Note: Group comparisons for self-reported (†) and validated abstinence (††), respectively: fully adjusted odds ratios (ORs), 95% confidence intervals (CIs): 12-month versus 3-month incentives (1.45 (0.86–2.43), *P* = 0.162; 1.15 (0.72–1.83), *P* = 0.561); 12-month incentives versus UC (1.60 (0.95–2.69), *P* = 0.075; 1.39 (0.87–2.23), *P* = 0.164); 3-month incentives versus UC (1.11 (0.95–2.69), *P* = 0.690; 1.21 (0.76–1.95), *P* = 0.420).
Abbreviations: iCO = single-person use carbon monoxide monitor; NA = not applicable; NRT = nicotine replacement therapy; SOS = significant other supporter.
[a]In no cases did verification test change outcome from abstinent to smoking.

Strengths and limitations

The high recruitment rate of 70%, together with no reports of study withdrawals, supports generalizability. Approximately three-quarters of participants were followed-up at 1-year postpartum, similar to the 6 months postpartum follow-up rate in a recent UK trial of incentives for smoking cessation during pregnancy [15]. Only approximately half of the target sample size was achieved; however, abstinence rates were higher than anticipated, which increased the power of the study, and our Bayesian approach suggested evidence for an effect. It may, however, still be underpowered, and this could explain the lack of significance for the comparison of 12-month incentives and UC. Our conservative approach of using a Bonferroni correction also contributed to the lack of significance for 12-month incentives versus UC. On reflection, considering the challenges of COVID and consequent reduced sample size, prior to commencing the analysis, we

13600443, 2024, 8, Downloaded from https://onlinelibrary.wiley.com/doi/10.1111/add.16487 by Test, Wiley Online Library on [17/10/2024]. See the Terms and Conditions (https://onlinelibrary.wiley.com/terms-and-conditions) on Wiley Online Library for rules of use; OA articles are governed by the applicable Creative Commons License

would have been justified in adopting a revised analysis plan. This could have included two primary analyses, comparing each incentive intervention with UC using $P < 0.05$, and a secondary analysis comparing the incentive interventions using a Bonferroni correction, in which case the results would have been definitive. Follow-up rates were highest in the 12-month incentives group, consistent with evidence that incentives improve retention [37]. This may have increased abstinence rates in this group, as missing data were analysed as smokers. However, this does not seem to explain the findings, as results were similar with alternative approaches to dealing with missing data.

For the primary outcome, rates of providing biochemical verification were lower in the 3-month incentive group compared with the other groups, which may have led to underestimation of the effect of this intervention. However, this is unlikely to have affected the findings for the primary outcome, as self-reports of abstinence mirror findings for the primary outcome, and the verification test changed the outcome from abstinent to not abstinent in only a few cases (see Table 2). Some baseline characteristics of those providing versus not providing verification were different, although there was little evidence to suggest that the missingness mechanism differed by randomized group (Supporting information, Data S3: C). The primary outcome was assessed unblinded, but as the assessment involved biochemical verification we consider there to be a low risk of bias. We neglected to register the trial protocol until five participants had been recruited. However, as there was only a slight delay in registration, only a few participants had been randomized and no substantive changes were made to the methods after the trial started, we consider that this does not affect the transparency, validity or reliability of the data. The findings are specific to England and to those having received incentives for smoking cessation during pregnancy; in England, from 2024, all pregnant women who smoke will be offered financial incentives to stop smoking. This limits generalizability to other smokers who have not taken part in incentive schemes to help them stop smoking during pregnancy. A final limitation is that almost all participants were of white ethnicity, although this is consistent with previous UK trials of incentives for smoking cessation during pregnancy [15, 29].

Comparisons with other studies

This was the first randomized controlled trial, to our knowledge, to examine whether offering postpartum financial incentives reduces postpartum smoking. It was also the first study to offer incentives up to 12 months after birth. Consistent with five trials of financial incentives for smoking cessation in pregnancy [17–21], postpartum incentives were considered acceptable by both participants and those offering the incentives (reported elsewhere, in the process evaluation). Consistent with one previous study providing postpartum incentives [17], the offer of incentives for a SOS was well received. Approximately half of participants recruited a SOS, with about half of these achieving an incentive payment.

Implications for policy and research

The findings suggest that providing postpartum incentives to women who have quit smoking during pregnancy can help to maintain long-term abstinence. However, our results suggest that incentives need to be retained during a long period. We found no benefit for abstinence for 3-months postpartum incentives. There is promising, but not definite, evidence for 12-months incentives. There was more than a two-fold increase in abstinence compared with the 3-month intervention, but the comparison with UC was not significant using a Bonferroni correction. However, at the upper confidence interval, this comparison suggested a possible two- to threefold benefit for the 12-month intervention and a point estimate suggesting an effect (OR = 1.67) that is likely to be clinically meaningful.

A definitive trial is needed to confirm whether an incentives intervention for 12 months postpartum is effective compared with UC. The evidence from the present study could provide an informative prior for a Bayesian trial design [38]. It is important to assess abstinence in the long term, after incentives are withdrawn, and we are assessing abstinence in this trial beyond 2 years postpartum. Research should examine which format and incentive level, at what frequency and duration achieves the most effective and cost-effective outcome [39] and whether the intervention is generalizable to women not receiving pregnancy incentives. Relapse rates were high in the first month and a more intensive initial intervention may be needed; a recent trial offered 16 incentives over 3 months postpartum with promising results [21]. We were unable to examine how the effect of social support from a SOS plus incentives compares with incentives alone, and this merits investigation. Research needs to explore the potential benefits of combining postpartum incentives with other behavioural interventions (e.g. [40]) and with pharmacological interventions. Approximately a third of participants had a partner who smoked and/or lived with smokers. Interventions need evaluating which target these individuals, such as having a partner or household member who smokes increases risk of postpartum relapse [8], and household smoke is harmful to children [6]. Little is known about triggers to smoking relapse during postpartum (e.g. stress of childcare, mood disturbance) [41, 42]; these triggers need to be clearly identified, so that interventions can target them and support behaviour change.

AUTHOR CONTRIBUTIONS

Michael Ussher: Conceptualization (lead); data curation (lead); formal analysis (supporting); funding acquisition (lead); investigation (lead); methodology (lead); project administration (lead); resources (lead); supervision (lead); validation (lead); writing—original draft (lead); writing—review and editing (lead). **Catherine Best:** Data curation (supporting); formal analysis (lead); investigation (supporting); methodology (supporting); project administration (supporting); validation (supporting); writing—original draft (supporting); writing—review and editing (supporting). **Sarah Lewis:** Formal analysis (supporting); methodology (supporting); writing—original draft (supporting); writing—review and editing (supporting). **Jennifer McKell:** Data curation (supporting);

13600443, 2024, 8, Downloaded from https://onlinelibrary.wiley.com/doi/10.1111/add.16487 by Test, Wiley Online Library on [17/10/2024]. See the Terms and Conditions (https://onlinelibrary.wiley.com/terms-and-conditions) on Wiley Online Library for rules of use; OA articles are governed by the applicable Creative Commons License

methodology (equal); validation (supporting); writing—review and editing (supporting). **Tim Coleman:** Investigation (supporting); methodology (supporting); writing—review and editing (supporting). **Sue Cooper:** Investigation (supporting); methodology (supporting); writing—review and editing (supporting). **Sophie Orton:** Investigation (supporting); methodology (supporting); writing—review and editing (supporting). **Linda Bauld:** Conceptualization (equal); funding acquisition (equal); investigation (supporting); methodology (supporting); writing—review and editing (supporting).

ACKNOWLEDGEMENTS

We thank the Greater Manchester Authority for Funding the study. We acknowledge the support of the senior management team for the Smokefree Pregnancy Programme at NHS Greater Manchester, including Jane Coyne, Andrea Crossfield and Fran Frankland. We thank the specialist maternity treating tobacco dependency teams across NHS Greater Manchester; without their help and drive to support families to remain smoke-free in pregnancy and postpartum recruitment to this study would not have been possible. We acknowledge the specific stop smoking service staff who delivered the interventions and conducted the research assessments: Joanne Pinder, Shelly Evans, Helen Parkinson, Wendy Whittle, Nicola Archer, Alex Smith, Joanne Buckley, Sharon Hall, Farzana Jabin, Lorraine Greathead, Andrea Robertson, Mandy Allanson, Jennifer Lowry, Elizabeth Warburton, Andrea Metcalfe, Samantha McNamara, Julie Brooks, Richard Waller, Joanne Collier, Claire Pollitt, Chris Bassett, Olwen Senogles, Nurun Begum and Marie Donovan. We acknowledge the stop smoking service managers: Northern Care Alliance NHS Foundation Trust: Andrea Robertson and Karen Clough; Bolton NHS Foundation trust: Andrea Metcalfe; Tameside and Glossop Integrated Care NHS Foundation Trust: Chris Bassett and Wendy Whittle; Manchester University NHS Foundation trust: Anita Luxford and Richard Waller. We thank Ana Howarth for assistance with data entry. We acknowledge the independent trial steering committee: Caitlin Notley (Chair), Allan Clark (Statistician), Jayne Coyne (funder representative), David Tappin, Jo Locker and Martyn Willmore. Tim Coleman is a National Institute for Health Research Senior Investigator.

DECLARATION OF INTERESTS

The funder had no role in considering the study design or in collection, analysis, interpretation of data, writing of the report or decision to submit for publication. The University of Stirling sponsored the study; the authors alone decided to publish the paper without influence from the Sponsor. There are no other competing interests to declare.

DATA AVAILABILITY STATEMENT

The datasets generated and analysed during the current study will be available on reasonable request to the chief investigator (Professor Michael Ussher, email: mussher@sgul.ac.uk) once the main findings of the trial have been accepted for publication, for up to 10 years. The researchers will decide whether to share data on an individual basis depending on the aims of the research and subject to a data sharing agreement. Individual de-identified participant data will be shared.

TRIAL REGISTRATION

ISRCTN Registry ISRCTN55218215. Date submitted for registration 6 June 2019; date registered 5 July 2019.

ORCID

Michael Ussher https://orcid.org/0000-0002-0995-7955
Jennifer McKell https://orcid.org/0000-0002-2912-0837
Tim Coleman https://orcid.org/0000-0002-7303-4805
Sophie Orton https://orcid.org/0000-0002-8577-216X

REFERENCES

1. Chamberlain C, O'Mara-Eves A, Porter J, Coleman T, Perlen SM, Thomas J, et al. Cochrane Pregnancy and Childbirth Group. Psychosocial interventions for supporting women to stop smoking in pregnancy. Cochrane Database Syst Rev. 2017;2:CD001055.
2. Simmons VN, Sutton SK, Quinn GP, Meade CD, Brandon TH. Prepartum and postpartum predictors of smoking. Nicotine Tob Res. 2014; 16:461–8.
3. Jones M, Lewis S, Parrott S, Wormall S, Coleman T. Re-starting smoking in the postpartum period after receiving a smoking cessation intervention: a systematic review. Addiction. 2016;111: 981–90.
4. Risch HA, Howe GR, Jain M, Burch JD, Holowaty EJ, Miller AB. Are female smokers at higher risk for lung cancer than male smokers? A case-control analysis by histologic type. Am J Epidemiol. 1993;138: 281–93.
5. Thun MJ, Carter BD, Feskanich D, Freedman ND, Prentice R, Lopez AD, et al. 50-year trends in smoking-related mortality in the United States. N Engl J Med. 2013;368:351–64.
6. Oberg M, Jaakkola MS, Woodward A, Peruga A, Prüss-Ustün A. Worldwide burden of disease from exposure to second-hand smoke: a retrospective analysis of data from 192 countries. Lancet. 2011; 377:139–46.
7. Leonardi-Bee J, Jere ML, Britton J. Exposure to parental and sibling smoking and the risk of smoking uptake in childhood and adolescence: a systematic review and meta-analysis. Thorax. 2011;66: 847–55.
8. Orton S, Coleman T, Coleman-Haynes T, Ussher M. Predictors of postpartum return to smoking: a systematic review. Nicotine Tob Res. 2018;20:665–73.
9. Livingstone-Banks J, Norris E, Hartmann-Boyce J, West R, Jarvis M, Chubb E, et al. Cochrane Tobacco Addiction Group. Relapse prevention interventions for smoking cessation. Cochrane Database Syst Rev. 2019;10:CD003999.
10. Kock LS, Erath TG, Coleman SRM, Higgins ST, Heil SH. Contingency management interventions for abstinence from cigarette smoking in pregnancy and postpartum: a systematic review and meta-analysis. Prev Med. 2023;31:107654.
11. National Institute of Health and Care Excellence, 2021. Tobacco: preventing uptake, promoting quitting and treating dependence. Recommendations on treating tobacco dependence in pregnant women. 2023. Available from: https://www.nice.org.uk/guidance/ng209/chapter/Recommendations-on-treating-tobacco-dependence-in-pregnant-women Accessed 13 Nov.
12. Too E, Hastie CE, McMeekin N, Lucas G, Tappin MD. Are financial incentives effective and cost-effective in a 'real life' smoking cessation program for pregnant women? A phase IV 'before and after' study to provide evidence to secure long-term funding. J Women's Health Reprod Med. 2021;5:17.
13. McMeekin N, Sinclair L, Robinson-Smith L, Mitchell A, Bauld L, Tappin DM, et al. Financial incentives for quitting smoking in pregnancy: are they cost-effective? Addiction. 2023;118:1445–56.

13600443, 2024, 8, Downloaded from https://onlinelibrary.wiley.com/doi/10.1111/add.16487 by Test, Wiley Online Library on [17/10/2024]. See the Terms and Conditions (https://onlinelibrary.wiley.com/terms-and-conditions) on Wiley Online Library for rules of use; OA articles are governed by the applicable Creative Commons License

14. Shepard DS, Slade EP, Nighbor TD, DeSarno MJ, Roemhildt ML, Williams RK, et al. Economic analysis of financial incentives for smoking cessation during pregnancy and postpartum. Prev Med. 2022;165:107079.
15. Tappin D, Sinclair L, Kee F, McFadden M, Robinson-Smith L, Mitchell A, et al. Effect of financial voucher incentives provided with UK stop smoking services on the cessation of smoking in pregnant women (CPIT III): pragmatic, multicentre, single blinded, phase 3, randomised controlled trial. BMJ. 2022;379:e071522.
16. UK Government, 2023. Pregnant Women Will be Offered Financial Incentives to Help Them Quit Smoking [internet] [cited 2023 Apr 10]. Available from: https://www.gov.uk/government/news/smokers-urged-to-swap-cigarettes-for-vapes-in-world-first-scheme Accessed 13 Nov 2023.
17. Donatelle RJ, Prows SL, Champeau D, Hudson D. Randomised controlled trial using social support and financial incentives for high risk pregnant smokers: significant other supporter (SOS) program. Tob Control. 2000;9:III67–9.
18. Heil SH, Higgins ST, Bernstein IM, Solomon LJ, Rogers RE, Thomas CS, et al. Effects of voucher-based incentives on abstinence from cigarette smoking and fetal growth among pregnant women. Addiction. 2008;103:1009–18.
19. Higgins ST, Washio Y, Lopez AA, Heil SH, Solomon LJ, Lynch ME, et al. Examining two different schedules of financial incentives for smoking cessation among pregnant women. Prev Med. 2014;68:51–7.
20. Higgins ST, Nighbor TD, Kurti AN, Heil SH, Slade EP, Shepard DS, et al. Randomized controlled trial examining the efficacy of adding financial incentives to best practices for smoking cessation among pregnant and newly postpartum women. Prev Med. 2022;165:107012.
21. Kurti AN, Nighbor TD, Tang K, Bolívar HA, Evemy CG, Skelly J, et al. Effect of smartphone-based financial incentives on Peripartum smoking among pregnant individuals: a randomized clinical trial. JAMA Netw Open. 2022;5:e2211889.
22. Ussher M, Best C, Lewis S, McKell J, Coleman T, Cooper S, et al. Financial incentives for preventing postpartum return to smoking (FIPPS): study protocol for a three-arm randomised controlled trial. Trials. 2021;22:512.
23. Wong HY, Subramaniyan M, Bullen C, Amer Siddiq AN, Danaee M, Yee A. The mobile-phone-based iCO™ Smokerlyzer®: comparison with the piCO+ Smokerlyzer® among smokers undergoing methadone-maintained therapy. Tob Induc Dis. 2019;17:65.
24. Bailey BA. Using expired air carbon monoxide to determine smoking status during pregnancy: preliminary identification of an appropriately sensitive and specific cut-point. Addict Behav. 2013;38:2547–50.
25. Bauld L, Hackshaw L, Ferguson J, Coleman T, Taylor G, Salway R. Implementation of routine biochemical validation and an 'opt out' referral pathway for smoking cessation in pregnancy. Addiction. 2012;107:53–60.
26. West R, Hajek P, Stead L, Stapleton J. Outcome criteria in smoking cessation trials: proposal for a common standard. Addiction. 2005;100:299–303.
27. Benowitz NL, Bernert JT, Foulds J, Hecht SS, Jacob P, Jarvis MJ, et al. Biochemical verification of tobacco use and abstinence: 2019 update. Nicotine Tob Res. 2020;22:1086–97.
28. von Weymarn LB, Thomson NM, Donny EC, Hatsukami DK, Murphy SE. Quantitation of the minor tobacco alkaloids nornicotine, anatabine, and anabasine in smokers' urine by high throughput liquid chromatography-mass spectrometry. Chem Res Toxicol. 2016;29:390–7.
29. Tappin D, Bauld L, Purves D, Boyd K, Sinclair L, MacAskill S, et al. Financial incentives for smoking cessation in pregnancy: randomised controlled trial. BMJ. 2015;350:h134.
30. Dumville JC, Torgerson DJ, Hewitt CE. Reporting attrition in randomised controlled trials. BMJ. 2006;332:969–71.
31. Kahan BC, Morris TP. Reporting and analysis of trials using stratified randomisation in leading medical journals: review and reanalysis. BMJ. 2012;345:e5840.
32. European Medicines Agency. Guideline on multiplicity issues in clinical trials. 2016. Available from: https://www.ema.europa.eu/en/documents/scientific-guideline/draft-guideline-multiplicity-issues-clinical-trials_en.pdf Accessed 13 Nov 2023.
33. Kahan BC, Jairath V, Doré CJ, Morris TP. The risks and rewards of covariate adjustment in randomized trials: an assessment of 12 outcomes from 8 studies. Trials. 2014;15:139.
34. Murray D, Cox JL. Screening for depression during pregnancy with the Edinburgh depression scale (EDDS). J Reprod Infant Psychol. 1990;8:99–107.
35. Fergusson D, Aaron SD, Guyatt G, Hébert P. Post-randomisation exclusions: the intention to treat principle and excluding patients from analysis. BMJ. 2002;325:652–4.
36. Johnston E, Bains M, Hunter A, Langley T. The impact of the COVID-19 pandemic on smoking, vaping, and smoking cessation Services in the United Kingdom: a qualitative study. Nicotine Tob Res. 2023;25:339–44.
37. Bricca A, Swithenbank Z, Scott N, Treweek S, Johnston M, Black N, et al. Predictors of recruitment and retention in randomized controlled trials of behavioural smoking cessation interventions: a systematic review and meta-regression analysis. Addiction. 2022;117:299–311.
38. Muehlemann N, Zhou T, Mukherjee R, Hossain MI, Roychoudhury S, Russek-Cohen E. A tutorial on modern Bayesian methods in clinical trials. Ther Innov Regul Sci. 2023;57:402–16.
39. Breen RJ, Palmer MA, Frandsen M, Ferguson SG. Design of Financial Incentive Programs for smoking cessation: a discrete choice experiment. Nicotine Tob Res. 2022;24:1661–8.
40. Notley C, Brown TJ, Bauld L, Clark AB, Duneclift S, Gilroy V, et al. BabyBreathe trial: protocol for a randomised controlled trial of a complex intervention to prevent postpartum return to smoking. BMJ Open. 2023;13:e076458.
41. Levine MD, Marcus MD. Do changes in mood and concerns about weight relate to smoking relapse in the postpartum period? Arch Womens' Ment Health. 2004;7:155–66.
42. Notley C, Blyth A, Craig J, Edwards A, Holland R. Postpartum smoking relapse--a thematic synthesis of qualitative studies. Addiction. 2015;110:1712–23.

SUPPORTING INFORMATION

Additional supporting information can be found online in the Supporting Information section at the end of this article.

How to cite this article: Ussher M, Best C, Lewis S, McKell J, Coleman T, Cooper S, et al. Effect of 3 months and 12 months of financial incentives on 12-month postpartum smoking cessation maintenance: A randomized controlled trial. Addiction. 2024;119(8):1352–63. https://doi.org/10.1111/add.16487

13600443, 2024, 8, Downloaded from https://onlinelibrary.wiley.com/doi/10.1111/add.16487 by Test, Wiley Online Library on [17/10/2024]. See the Terms and Conditions (https://onlinelibrary.wiley.com/terms-and-conditions) on Wiley Online Library for rules of use; OA articles are governed by the applicable Creative Commons License
