# Supplementary material for: Financial incentives for maintaining postpartum smoking abstinence: 3–4‐year follow‐up of the Financial Incentives for Prevention of Postpartum Return to Smoking (FIPPS) randomised controlled trial
Source: Addiction. 2026 Jul 14;121(10):2725–34. doi: 10.1111/add.70505 (PMC13578832; doi:10.1111/add.70505)
Supplement: Supplementary file 3 [file ADD-121-2725-s001.docx]

**Supplementary Information C**

**STUDY & DATA MONITORING PLAN**

**F**inancial **I**ncentives for **P**reventing **P**ostpartum return

to **S**moking (FIPPS)

Version 1.0	February 1^st^, 2019

**CHIEF INVESTIGATOR**

Professor Michael Ussher

Institute of Social Marketing and Health

University of Stirling

Stirling

FK9 4LA

**Phone:** 0778 0662 8572

**Email:** mussher@sgul.ac.uk

| **SPONSOR**  University of Stirling | **SPONSOR REPRESENTATIVE**  Ms Rachel Beaton  Research and Development  University of Stirling,  Stirling FK9 4LQ, UK  E-mail joy.taylor@stir.ac.uk  Telephone 01786466443 |
| --- | --- |
|  | **Phone:** 01786 466443 |
|  | **Email:** rachel.beaton@stir.ac.uk |

**REFERENCE NUMBERS**

ISRCTN: ISRCTN55218215 - registered 5^th^ July 2019

IRAS Project ID: 227489

REC Ref: 18/NW/0838 North West - Liverpool Central Research Ethics Committee

**Contents**

**1. Aim of study and data monitoring plan	3**

**2. Persons responsible for conducting monitoring	3**

**3. Risk assessment	3**

**4. Data quality assurance	3**

**5. Remote or on-site data monitoring	3**

5.1 Data transfer	3

5.2 Nature and frequency of site-specific monitoring	3

5.3 Site self-data monitoring	4

5.4 Data summaries	4

**6. Source data verification	4**

6.1 Screening/eligibility	4

6.2 Consent	5

6.3 Data entry for participants assessments	5

6.4 Saliva sample labels	5

**1. Aim of study and data monitoring plan**

The purpose of this plan/document is to outline the monitoring requirements and data quality assurance for the FIPPS study.

**2. Persons responsible for conducting monitoring**

Monitoring and/or audits will be conducted by the trial coordinator and trial researchers at University of Stirling. The study may also be subject to inspection by the funders and/or inspection and audit by the sponsor.

**3. Risk assessment**

A formal risk assessment has not been conducted by the sponsor as the trial is a non-CTIMP study and participant recruitment and the interventions are of minimal risk. The main risk is disclosure of identifiable data.

**4. Data quality assurance**

The study will be fully compliant with the Research Governance Framework. Audits of enrolment, participant retention and outcome assessment will be performed by the trial co­ordinator. Written reports will be produced for the Trial Steering Committee (TSC to take the role of the Data Monitoring Committee) informing them of any corrective action that is required.

**5. Remote or on-site data monitoring**

Data monitoring includes that conducted remotely or on-site by trial coordinator and trial researchers at University of Stirling.

The purpose of this monitoring is to:

- Assess protocol compliance and ensure trial procedures are being followed.
- Check use of current trial documents.
- Verify research data to ensure accuracy and completeness against source documents.
- Ensure accurate entry of information on saliva specimen labels.

**5.1 Data transfer**

All trial data (e.g., screening forms/logs, consent forms/logs, baseline and follow-up assessment data) will be sent securely, on a weekly basis, from the sites to the trial coordinator. Baseline and follow-up assessment data will be collected on paper or electronic forms and the forms will be sent securely to the trial coordinator.

**5.2 Nature and frequency of site-specific monitoring**

Comprehensive data monitoring (on-site or remote), covering all data and documentation, will be conducted at least once to capture each stage of data collection and on an ‘as-needed’ basis thereafter and dependent on factors such as:

- Recruitment rates.
- If there are any concerns reported by sites which may indicate the integrity of randomisation or data collection may be compromised.
- The amount of data identified as inaccurate/missing during the first data monitoring event.
- If a stage of data collection (e.g., recruitment, three months postpartum, 12 months postpartum) was not captured during the first data monitoring event.

Where an on-site visit is not deemed necessary, monitoring will be completed remotely over the phone or videocall between the trial coordinator and each site. Prior to a comprehensive data monitoring event, the site will be asked to send the trial coordinator the delegation log, screening log, consent log and Electronic Investigator Site File. The trial coordinator will provide the site with a list of the data that will be checked. The monitoring event will include source data verification (i.e., checking trial data against source data - see section 7. below) and examining the investigator Site file to check that current documents and procedures are being used; check CVs and GCP certificates are on file; and check that delegation log is on file and is complete. Documentation and data will be checked for completeness, accuracy, and legibility. A ‘Monitoring Summary Report’ will be completed following comprehensive monitoring event and, if necessary, an action list completed.

In addition, to comprehensive data monitoring events, screening, consent and participant assessment data will be checked on weekly basis.

**5.3 Site self-data monitoring**

Site-self monitoring is to ensure that the study is being run and documented to a standard that would be acceptable to being audited by the local site R&D Department. We propose that sites complete self-monitoring at different stages throughout the trial to ensure all data collection points have been checked at least once. Sites will provide self-monitoring feedback to the trial coordinator.

**5.4 Data summaries**

Summary reports required for data monitoring and data quality assurance will be generated by the trial coordinator, on an ‘as needed’ basis, for the following data:

- Numbers screened
- Number/proportion eligible and ineligible
- Number/proportion eligible and not interested in participating
- Number/proportion consented
- Number/proportion randomised
- Number/proportion completing follow-up at three months postpartum
- Number/proportion completing follow-up at 12 months postpartum
- Number of participants receiving vouchers at each time point
- Number of significant other supporters receiving vouchers
- Number withdrawn from trial

**6. Source data verification**

Source data verification involves checking to ensure a high level of data quality and integrity in the final dataset available for analysis. Data recorded in the trial databases will be checked for consistency with the source data recorded on electronic or paper forms at each site.

**6.1 Screening/eligibility**

On a weekly basis, 100% of screening forms will be checked to ensure that they have been fully completed, that the eligibility criteria have been satisfied and that the data in the screening form matches the screening log, and errors will be corrected. If the screening form shows that a participant was randomised when they weren’t eligible, the site will be contacted to check whether there is an error on the form. If it is confirmed that the participant is ineligible, the trial steering committee will be consulted (blind to study group) to decide whether this person should be withdrawn from the trial and their data not included in the analysis.

The version number of the screening form will also be checked. If an older version was used the site will be alerted and the site may need to contact the participant to check they are still eligible based on the current screening form.

**6.2 Consent**

On a weekly basis, 100% of consent forms will be checked for full completion and for current version number. If the form is incomplete (e.g., not signed) or an older version number is used the site will need to contact the individual to complete form. 100% of forms will also be checked against the consent log and errors will be corrected and fed back to the site.

**6.3 Data entry for participant assessments**

On an, at least, monthly basis, for participant assessments at baseline, three 12 months postpartum, and anu further long-terms follow-ups, 100% of paper or electronic questionnaires will be checked for version number, missing data, and data out of the expected range. The site may need to be directed to contact the participant to correct errors or add missing data.

For all assessments, data from the questionnaires will be manually entered into an SPSS database by a researcher at University of Stirling.

For baseline assessments, 20% of participants data in the database will be randomly selected and checked against the source questionnaires by a second researcher. If there is more than 3% data entry error then all the data will be checked.

For assessments at three months, 12 months postpartum, and anu further long-term assessments 100% of the data entry will be checked by a second researcher.

**6.4 Saliva sample labels**

As early as possible in the study, at each site, one saliva sample label completed by each stop smoking advisor will be checked to ensure that the label is completed accurately. If a label is found to have an error (e.g., incorrect participant ID), then all the labels for the advisor will be checked and corrected.
