# Supplementary material for: Financial incentives for maintaining postpartum smoking abstinence: 3–4‐year follow‐up of the Financial Incentives for Prevention of Postpartum Return to Smoking (FIPPS) randomised controlled trial
Source: Addiction. 2026 Jul 14;121(10):2725–34. doi: 10.1111/add.70505 (PMC13578832; doi:10.1111/add.70505)
Supplement: Supplementary file 4 [file ADD-121-2725-s002.docx]

| **Supporting Information E. Among those self-reporting as non-smokers at 3-4 years, baseline characteristics of participants undergoing a test of exhaled carbon monoxide (CO) as verification of smoking status, and of participants not undergoing this test, by study group**  Summary of comparison: The characteristics of those providing and not providing verification are similar except those providing verification tended to report lower levels of education; increased likelihood of white ethnicity and of using nicotine replacement therapy; decreased likelihood of having a significant other supporter and of having a major depressive disorder. However, there was little evidence to suggest the missingness mechanism differed by randomised group.  **Data are number (%) of participants unless otherwise stated** | | | | | | | | | |
| --- | --- | --- | --- | --- | --- | --- | --- | --- | --- |
|  | **Participants undergoing CO test** | | | |  | **Participants not undergoing CO test** | | |  |
|  | Usual care (n=11) | 3-month incentives (n=19) | 12-month incentives (n=26) | | Total  (n=56) | Usual care (n=6) | 3-month incentives (n=11) | 12-month incentives (n=15) | Total  (n=32) |
| **Demographic characteristics** |  |  |  | |  |  |  |  |  |
| Maternal age in years Mean (SD) | 27.7 (5.3) | 28.9 (5.6) | 31.8 (4.5) | | 30.0 (5.3) | 34.8 (5.0) | 29.5 (4.7) | 26.7 (5.9) | 29.2 (6.0) |
| A-level, degree or equivalent | 5 (45.5) | 8 (42.1) | 18 (69.2) | 31 (55.4) | | 6 (100) | 7 (63.6) | 8 (53.3) | 21 (65.6) |
| White ethnicity | 11 (100.0) | 17 (89.5) | 25 (96.2) | | 53 (94.6) | 6 (100) | 8 (72.7) | 13 (86.7) | 27 (84.4) |
| **Type of stop smoking service**  Midwife led | 11 (100.0) | 18 (94.7) | 26 (100.0) | | 55 (98.2) | 6 (100) | 11 (100) | 13 (86.7) | 30 (93.8) |
| **Smoking history** |  |  |  | |  |  |  |  |  |
| Pre-pregnancy cigarettes smoked a day Mean (SD) | 11.3 (6.4) | 12.0 (5.7) | 12.8 (6.9) | | 12.2 (6.3) | 13.7 (6.8) | 16.2 (9.7) | 11.9 (5.7) | 13.7 (7.5) |
| Weeks of continuous smoking abstinence Mean (SD) | 24.9 (5.6) | 24.7 (4.5) | 24.3 (5.7) | | 24.6 (5.2) | 23.7 (4.7) | 20.3 (5.3) | 21.6 (7.0) | 21.5 (6.0) |
| Expired CO level ppm  Mean (SD) | 1.0 (0.8) | 0.8 (0.6) | 1.2 (0.9) | | 1.0 (0.8) | 1.2 (0.7) | 1.5 (0.8) | 1.3 (0.9) | 1.3 (0.8) |
| Very or extremely confident in maintaining smoking abstinence | 7 (63.6) | 10 (52.6) | 21 (80.8) | | 38 (67.9) | 5 (83.3) | 8 (72.7) | 10 (66.7) | 23 (71.9) |
| Uses nicotine replacement therapy | 1 (9.1) | 4 (21.1) | 4 (15.4) | | 9 (16.1) | 0 | 2 (18.2) | 0 | 2 (6.3) |
| Uses electronic-cigarettes | 5 (45.5) | 8 (42.1) | 9 (34.6) | | 22 (39.3) | 1 (16.7) | 5 (45.5) | 6 (40.0) | 12 (37.5) |
| Uses heat-not-burn | 1 (9.1) | 0 | 1 (3.8) | | 2 (3.6) | 0 | 0 | 0 | 0 |
| Uses of smoking cessation support beyond that in trial | 0 | 0 | 0 | | 0 | 0 | 0 | 0 | 0 |
| Partner smokes | 4 (36.4) | 3 (15.8) | 5 (19.2) | | 12 (21.4) | 0 | 4 (36.4) | 3 (20.0) | 7 (21.9) |
| Living with smokers | 4 (36.4) | 2 (10.5) | 6 (23.1) | | 12 (21.4) | 0 | 4 (36.4) | 4 (26.7) | 8 (25.0) |
| Has significant-other-supporter | 3 (27.3) | 9 (47.4) | 15 (57.7) | | 27 (48.2) | 5 (8.3) | 6 (54.5) | 10 (66.7) | 21 (65.6) |
| **Pregnancy** |  |  |  | |  |  |  |  |  |
| Weeks pregnant Mean (SD)^a^ | 36.5 (1.5) | 36.9 (1.4) | 36.3 (1.1) | | 36.6 (1.2) | 37.0 (1.4) | 35.9 (0.6) | 36.8 (1.4) | 36.6 (1.2) |
| Days postpartum Mean (SD)^b^ | 7.0 (3.6) | 8.0 (8.5) | 2.5 (2.1) | | 6.0 (4.8) | NA | 7.0 (2.8) | 7.0 (NA) | 7.0 (2.0) |
| Parity Mean (SD) | 1.5 (1.4) | 1.0 (1.1) | 1.4 (1.3) | | 1.3 (1.2) | 0.7 (1.3) | 2.4 (2.8) | 0.9 (0.9) | 1.4 (1.9) |
| Intend to bottle and breastfeed | 1 (9.1) | 3 (15.8) | 5 (19.2) | | 9 (16.1) | 3 (50.0) | 1 (9.1) | 4 (26.7) | 8 (25.0) |
| Intend to breastfeed only | 4 (36.4) | 8 (42.1) | 16 (61.5) | | 28 (50.0) | 3 (50.0) | 7 (63.6) | 4 (26.7) | 14 (43.8) |
| Intend to breastfeed for ≥ 4 months | 2 (40.0) | 2 (18.2) | 14 (66.7) | | 18 (48.6) | 2 (33.3) | 5 (62.5) | 3 (42.9) | 10 (47.6) |
| **Alcohol Use Disorders Identification-Consumption** |  |  |  | |  |  |  |  |  |
| Risk drinking (score ≥ 5) | 0 | 0 | 0 | | 0 | 0 | 1 (9.1) | 1 (6.6) | 2 (6.3) |
| **Edinburgh Depression Scale** (EDS) |  |  |  | |  |  |  |  |  |
| Overall EDS scores Mean (SD) | 6.6 (6.8)^m[1]^ | 5.1 (3.9) | 6.2 (3.9)^m[1]^ | | 5.9 (4.5)^m[2]^ | 5.5 (5.7) | 6.2 (6.0) | 4.3 (4.8) | 5.2 (5.2) |
| Major Depressive Disorder  (EDS ≥11) | 2 (20.0)^m[1]^ | 2 (10.5) | 4 (16.0)^m[1]^ | | 8 (14.8)^m[2]^ | 2 (33.3) | 3 (27.3) | 3 (20.0) | 8 (25.0) |

^m[ ]^ Missing data ^[number missing]^, SD=standard deviation, CO=carbon monoxide, ppm=parts per million, NA=not applicable, ^a^relates to those recruited in pregnancy, ^b^relates to those recruited postpartum
