# Supplementary material for: Financial incentives for maintaining postpartum smoking abstinence: 3–4‐year follow‐up of the Financial Incentives for Prevention of Postpartum Return to Smoking (FIPPS) randomised controlled trial
Source: Addiction. 2026 Jul 14;121(10):2725–34. doi: 10.1111/add.70505 (PMC13578832; doi:10.1111/add.70505)
Supplement: Supplementary file 5 [file ADD-121-2725-s004.docx]

**Supporting information-G: Multiple imputation**

Multiple imputation was by chained equations and was conducted in Stata version 17. Imputed variables were: the outcome biochemically verified abstinence at 3yrs, and in addition outcome at 12 months, age, very or extremely confident in ability to remain abstinent at baseline, cigarettes per day pre pregnancy, depression score at baseline, weeks since last puff at baseline, educated to degree level. Other variables without missingness used as auxiliary variables in the imputation were whether there was a smoker in the home at baseline, whether their partner smoked, white versus other ethnic group, randomisation group and site. Binary variables were imputed with ‘logit’ and continuous variables with ‘regress’.

1000 imputed dataset were created and then analysed using a logistic regression with randomisation group as independent variable and site as covariate. The parameters obtained were combined using Rubin’s rules.

**Conclusion**: Under a missing at random assumption which assumes missingness is non-informative conditional on the observed variables, and therefore imputes the outcome with the conditional probability observed in the non-missing cases-there is no difference between the no incentives group and the other group groups in the odds of abstinence at 12 month follow up. Under these assumptions there is also no difference between the 3 month incentives group and the 12 month incentives group.
