# Supplementary material for: Financial incentives for maintaining postpartum smoking abstinence: 3–4‐year follow‐up of the Financial Incentives for Prevention of Postpartum Return to Smoking (FIPPS) randomised controlled trial
Source: Addiction. 2026 Jul 14;121(10):2725–34. doi: 10.1111/add.70505 (PMC13578832; doi:10.1111/add.70505)
Supplement: Supplementary file 6 [file ADD-121-2725-s005.docx]

**Supplementary Information H: Pattern mixture models**

Pattern mixture models model the data of interest jointly with the assumed missing data mechanism (White et al 2018). We defined sensitivity parameters for the degree of departure from the missing at random assumption (under which the conditional expectation of the outcomes for which data is missing is the same as those for which we observe data) and analysed the primary outcome under the following variations from the base case: non-responders not all missing are smokers, non-responders in one group are not all smokers, non-responders in the other group are not all smokers.

The graphs below illustrate the results of missing data sensitivity analysis using the pattern mixture model approach, for each of the three group comparisons. The graphs demonstrate how the adjusted odds ratio of being a biochemically-verified non-smoker varies for varying values of the informative missingness odds ratio in each of the study groups and for the groups combined (i.e. varying amounts of deviation from the missing at random assumption). Sensitivity is on the base case of all missing are smokers (OR for missingness on outcome=0), shown on far left of graphs.

The analysis was based on a cross-sectional comparison of the intervention groups at 3-4 year follow up.

White IR, Carpenter J, Horton NJ. A mean score method for sensitivity analysis to departures from the missing at random assumption in randomised trials. Stat Sin. 2018 Oct;28(4):1985-2003.

The multiple imputation was conducted in Stata using the ‘mi impute chained’ command. The variables included in the imputation were binary variables: outcome at 3-4 year follow up (with lost to follow up as missing), outcome at 12 months, Very or extremely confident in maintaining smoking abstinence, education to A-level, degree or equivalent, continuous variables: age, cigarettes per day pre-pregnancy, depression score, weeks since last smoked at baseline. Variables included in the imputation that did not have any missing data included whether there was a smoker in the home, whether partner smoked, ethnic group (white versus other group) and randomisation group. A chain-only imputation was conducted to check the stability of the trace before the main imputation was conducted. A thousand imputations were conducted. The ‘mi estimate: logistic’ estimation command included centre as fixed effect as not possible to estimate random effects. The estimation model included only randomisation group and centre as independent variables.

The pattern mixture model was conducted in Stata using the ‘rctmiss’ command. The comparisons were conducted pairwise (12months incentives versus no incentives and 3 months incentives versus no incentives then a comparison of the two incentives groups). Syntax used was ‘rctmiss, sens(Rand_grp) pmmdelta(0(0.1)1, expdelta base(0)): logistic …’ Centre was included as a fixed effect as again not possible to estimate random effects.

No incentives (control) versus 3-month incentives

In the comparison of 3 months incentives group to the no incentives group the direction of effect under the base case of missing=smoking (purple line in graph) is that 3 months incentives is associated with a higher odds of abstinence than no incentives although this difference is not statistically significant. Under the condition that the missing= smoking assumption is relaxed for the 3m incentive group only (blue line in the graph) then the difference between groups is statistically significant. For the other condition where the missing =smoking assumption is relaxed for the no incentives group only (the red line in the graph) then there is no difference between groups.

No incentives versus 12 months incentives

Sensitivity on the base case of all missing are smokers (OR for missingness on outcome ==0). Under the missing =smoking case, which is shown on the far left of graph there is a difference between groups in the odds of smoking at 3-4yrs. If the missing=smoking assumption is relaxed for the 12 months incentives group only (blue line) then the difference between groups increases. If the missing=smoking assumption is relaxed for both groups (purple line) or for the no incentives group only (red line) then the difference between groups on the primary outcome decreases.

3-month incentives versus 12-month incentives

In the comparison of 3 months incentives to the 12-months incentives the direction of effect under the base case of missing=smoking (purple line in graph) is that 12 months incentives is associated with higher odds of abstinence than 3-month incentives although this difference is not statistically significant. Under the condition that the missing= smoking assumption is relaxed for the 12-months incentive group only (blue line in the graph) then the difference between groups is statistically significant but only once exp(delta) is 0.6, so when missing cases assumed more likely to be abstinent in the 12-month group. For the other condition where the missing =smoking assumption is relaxed for the 3-month incentives group only (the red line in the graph) then there is no difference between groups and this is also the case where the assumption is relaxed for both groups.
