# Supplementary material for: Financial incentives for maintaining postpartum smoking abstinence: 3–4‐year follow‐up of the Financial Incentives for Prevention of Postpartum Return to Smoking (FIPPS) randomised controlled trial
Source: Addiction. 2026 Jul 14;121(10):2725–34. doi: 10.1111/add.70505 (PMC13578832; doi:10.1111/add.70505)
Supplement: Supplementary file 7 [file ADD-121-2725-s006.docx]

**Supplementary Information F:** testing for a linear trend in abstinence across the three groups

There was a significant linear trend in abstinence across the three groups OR = 1.56 (1.09-2.23), P = 0.016.

We inspected the Information Criteria for the analysis with the independent variable (randomisation group) as a linear predictor versus in the primary analysis where it is dummy coded.

| Specification of independent variable (randomisation group) | df | Bayesian Information Criterion | Akaike's Information Criterion | Log likelihood |
| --- | --- | --- | --- | --- |
| Dummy coded (primary analysis) | 4 | 355.27 | 338.73 | -165.36 |
| Linear | 3 | 349.32 | 336.91 | -165.46 |

The Information Criteria are lower, indicating a better model fit for the linear model versus the dummy coded model, probably due to the lower complexity of the linear model, it having one fewer parameter.

Inspection of the Pearson residuals from the fixed part of the linear model does not indicate any major deviance from horizontal mean 0 as shown in the graph below. Together this is reassuring that the linear model is a reasonable fit to the data.
