# Supplementary material for: Financial incentives for maintaining postpartum smoking abstinence: 3–4‐year follow‐up of the Financial Incentives for Prevention of Postpartum Return to Smoking (FIPPS) randomised controlled trial
Source: Addiction. 2026 Jul 14;121(10):2725–34. doi: 10.1111/add.70505 (PMC13578832; doi:10.1111/add.70505)
Supplement: Supplementary file 8 [file ADD-121-2725-s008.docx]

 Trust logo here

**Supplementary Information D**

**F**inancial **I**ncentives for **P**reventing **P**ostpartum return to **S**moking

**Questionnaire at 3-4 years follow-up**

Version 1.0, October 20^th^, 2023

| **Trust**  **(circle box)** | NCA | MFT | Bolton | T&G |
| --- | --- | --- | --- | --- |

| **Smokefree programme no.** | P | 7 | 4 | 6 |
| --- | --- | --- | --- | --- |

| **Randomisation**  **Group**  (Circle box) | **No**  **Incentives**  **1** | **3**  **Months**  **2** | **12**  **Months**  **3** |
| --- | --- | --- | --- |

| **Randomisation**  **Number** |  |  |  |  |
| --- | --- | --- | --- | --- |

| **Participant initials** |  |  |  |
| --- | --- | --- | --- |
| **Stop smoking service advisor initials** |  |  |  |

| **Today’s Date** |  |  |  |
| --- | --- | --- | --- |

Day	month	year

I’d like to ask you a few questions about how you have been getting on since we last contacted you about this research study

**First, I’d like to ask about your smoking**

Have you smoked at all since we last contacted you about this research study?

(Please tick one response)

☐ No, not even a puff

☐ Yes, just a few puffs

☐ Yes, between 1 and 5 cigarettes

☐ Yes, more than 5 cigarettes

If ‘No, please arrange a time to meet face-to-face (most likely at woman’s home) to take an expired carbon monoxide reading, to check smoking status

Please enter the carbon monoxide reading here: ppm

**Next, I’d like to ask about any stop smoking aids you have used recently**

Have you used any **Nicotine Replacement Therapy,** such as a patch, in the last week?

Yes ☐ No ☐

If Yes, which types of Nicotine Replacement Therapy have you used? (Tick all that apply)

☐ Patch	☐ Lozenge	☐ Mouth Spray

☐ Gum	☐ Microtabs

☐ Inhalator	☐ Nasal Spray

Have you used an e-cigarette/vape in the last week? Yes ☐ No ☐

If Yes, would you like to try to stop vaping in the next few months? Yes ☐ No ☐

**Now, I’d like to ask about other smokers around you**

Does anyone living in your home smoke tobacco? Yes ☐ No ☐

If you have a partner, does your partner smoke tobacco? Yes ☐ No ☐ Not applicable ☐

**Next, I have a question about Breastfeeding**

Did you breastfeed the baby you gave birth to at the start of this research study?

Yes ☐ No ☐

If Yes – For how long did you breastfeed? (*Tick one only*):

**☐** First 1 to 2 weeks

**☐** 2 to 4 weeks

**☐** 1 to 2 months

☐ 2 to 3 months

**☐** 4 to 6 months

**☐** Over 6 months

Have you given birth again since the baby you had at the start of this research study?

Yes ☐ No ☐

Are you currently pregnant? Yes ☐ No ☐

Since you were last asked about your smoking for this research study, have you received any professional help with stopping smoking, such as from the Greater Manchester Smokefree Pregnancy programme?

Yes ☐ No ☐ If Yes, which organisation gave you help?

Please enter organisation name here:

***Thank you for helping us with our research by completing this questionnaire***

***If they report having smoked***

**I will give or send you a £20 love2shop voucher to thank you for helping us.**

***If they have not smoked and a carbon monoxide reading needs to be taken:***

**Once we have met to take a carbon monoxide reading, I will give you a £20 voucher to thank you for helping us. I’ll give you the voucher regardless of what the reading is*.***
